# RNA modifications: molecular orchestrators of wound healing

**DOI:** 10.1093/burnst/tkag010

**Published:** 2026-01-15

**Authors:** Xiuying Guo, Lele Liu, Junqi Yang, Yuhe Dai, Qianbo Zhang, Rifang Gu, Min Tan, Ming Tang, Xuqiang Nie

**Affiliations:** College of Pharmacy, Key Laboratory of the Basic Pharmacology of the Ministry of Education and Joint International Research Laboratory of Ethnomedicine of Ministry of Education, Zunyi Medical University, No. 6 Xuefu West Road, Xinpu New District, Zunyi City, Guizhou Province 563006, China; College of Pharmacy, Key Laboratory of the Basic Pharmacology of the Ministry of Education and Joint International Research Laboratory of Ethnomedicine of Ministry of Education, Zunyi Medical University, No. 6 Xuefu West Road, Xinpu New District, Zunyi City, Guizhou Province 563006, China; College of Pharmacy, Key Laboratory of the Basic Pharmacology of the Ministry of Education and Joint International Research Laboratory of Ethnomedicine of Ministry of Education, Zunyi Medical University, No. 6 Xuefu West Road, Xinpu New District, Zunyi City, Guizhou Province 563006, China; College of Pharmacy, Key Laboratory of the Basic Pharmacology of the Ministry of Education and Joint International Research Laboratory of Ethnomedicine of Ministry of Education, Zunyi Medical University, No. 6 Xuefu West Road, Xinpu New District, Zunyi City, Guizhou Province 563006, China; College of Pharmacy, Key Laboratory of the Basic Pharmacology of the Ministry of Education and Joint International Research Laboratory of Ethnomedicine of Ministry of Education, Zunyi Medical University, No. 6 Xuefu West Road, Xinpu New District, Zunyi City, Guizhou Province 563006, China; School Medical Office, Zunyi Medical University, No. 6 Xuefu West Road, Xinpu New District, Zunyi City, Guizhou Province 563006, China; College of Pharmacy, Key Laboratory of the Basic Pharmacology of the Ministry of Education and Joint International Research Laboratory of Ethnomedicine of Ministry of Education, Zunyi Medical University, No. 6 Xuefu West Road, Xinpu New District, Zunyi City, Guizhou Province 563006, China; Department of Structural Biology, St. Jude Children’s Research Hospital, 262 Danny Thomas Place, Memphis, TN 38105, United States; College of Pharmacy, Key Laboratory of the Basic Pharmacology of the Ministry of Education and Joint International Research Laboratory of Ethnomedicine of Ministry of Education, Zunyi Medical University, No. 6 Xuefu West Road, Xinpu New District, Zunyi City, Guizhou Province 563006, China; Guizhou Key Laboratory of Modern Traditional Chinese Medicine Creation, Zunyi Medical University, No. 6 Xuefu West Road, Xinpu New District, Zunyi City, Guizhou Province 563006, China; Zunyi Center for Disease Control and Prevention, No. 2 Xinlong Avenue, Xinpu New District, Honghuagang District, Zunyi 563000, Guizhou Province, China

**Keywords:** *N* ^6^-methyladenosine, 5-methylcytosine, *N* ^7^-methylguanosine, *N* ^4^-acetylcytidine, Wound healing, Fracture healing, Corneal repair, Epitranscriptomics

## Abstract

Wound healing is a highly coordinated biological process traditionally divided into three phases: inflammatory, proliferative, and remodeling. Diabetes and acute trauma markedly disrupt these stages, resulting in delayed wound closure, persistent inflammation, and impaired tissue regeneration. This review focuses on three trauma-relevant contexts: (i) skin wounds, including diabetic ulcers and burns; (ii) bone fracture healing; and (iii) corneal epithelial and stromal injury. Robust *in vivo* evidence is synthesized to delineate the mechanistic roles of the four principal ribonucleic acid (RNA) modifications: *N*^6^-methyladenosine, 5-methylcytosine, *N*^7^-methylguanosine, and *N*^4^-acetylcytidine. Additionally, the roles of RNA modification writers, erasers, and readers in regulating macrophage polarization, stem and progenitor cell fate, angiogenesis, lymphangiogenesis, and extracellular matrix remodeling are examined. Evidence across different tissues and wound healing phases is integrated rather than presented descriptively. Methodological limitations are highlighted, and knowledge gaps are identified alongside testable hypotheses. Translational opportunities with direct relevance to burn and trauma management are emphasized. This review aims to integrate mechanistic and translational insights into a coherent framework for therapeutic intervention. By defining how RNA modifications intersect with distinct wound healing phases, concrete therapeutic entry points and delivery strategies relevant to burns and trauma are identified, including topical hydrogels, exosome-based therapies, and bone-targeted nanoparticles. Designs for pragmatic clinical trials and biomarker strategies that enable translation of preclinical findings to patients are also discussed.

## Graphical Abstract

**Graphical Abstract:**
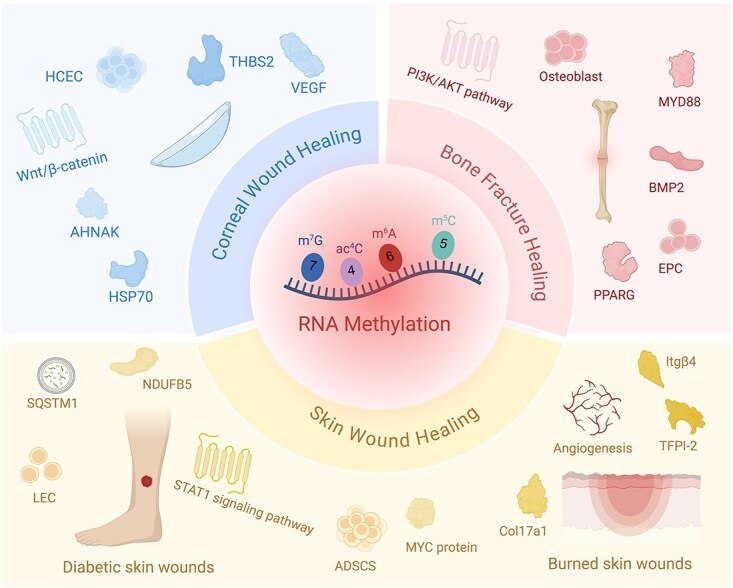

Figure created with BioRender.com (license: CP296TZDNK).

## Highlights

- *N* ^6^-methyladenosine (m^6^A) regulates keratinocyte and fibroblast migration/proliferation across diabetic, burn, and chronic wounds.
- METTL3/FTO-driven m^6^A dynamics control osteogenesis and fracture repair via SMAD5 and NF-κB signaling.
- m^6^A writers and readers, including METTL3 and YTHDF3, govern corneal epithelial regeneration and transparency.
- Additional RNA marks—m^5^C, m^7^G, ac^4^C, m^1^ψ—emerge as key regulators of angiogenesis and stem-cell–mediated repair.
- Organoid and 3D skin models facilitate translational studies of epitranscriptomic therapies for wound healing.

## Background

Chemical modifications of biological macromolecules are dynamic processes that critically determine molecular fate and function. Ribonucleic acid (RNA) modifications constitute a sophisticated layer of post-transcriptional regulation. These modifications involve the chemical tagging of RNA bases or the ribose moiety with spatiotemporal specificity, thereby profoundly influencing RNA metabolism, including stability, protein recruitment, translation, and splicing [1–3].

To date, more than 170 distinct RNA modifications have been characterized, including *N*^6^-methyladenosine (m^6^A), 5-methylcytosine (m^5^C), *N*^1^-methyladenosine (m^1^A), *N*^7^-methylguanosine (m^7^G), *N*^4^-acetylcytidine (ac^4^C), and pseudouridylation [4–6]. The dynamics of these modifications are regulated by specific proteins, classified as ‘writers’ (methyltransferases), ‘erasers’ (demethylases), and ‘readers’ (binding proteins). Erasers remove methylation marks to enable demethylation; writers install methylation on RNA; and readers recognize the modification to mediate downstream effects, including translation or RNA degradation [7–9].

Wound healing is a fundamental biological process that maintains tissue integrity and function. It progresses through overlapping yet distinct stages, including inflammation, cell proliferation and migration, differentiation, and extracellular matrix (ECM) remodeling [10]. Historically, research has concentrated on proteins, growth factors, and canonical signaling pathways, while epitranscriptomic regulation has recently emerged as a pivotal layer of control. Epitranscriptomics has demonstrated that RNA chemical modifications play a central role in regulating dynamic gene expression [11, 12]. As reversibly dynamic post-transcriptional regulators, RNA modifications modulate RNA stability, spatial localization, splicing efficiency, and translational activity, thereby contributing to diverse biological processes, such as cell fate programming, immune response modulation, and tissue regeneration [13–15].

m^6^A represents the most prevalent post-transcriptional modification of eukaryotic messenger RNA (mRNA). Its regulatory functions enhance wound healing [14, 16]. A study has reported that m^6^A methylation precisely orchestrates key biological processes within the wound microenvironment, including modulation of inflammatory responses, promotion of angiogenesis, and optimization of collagen remodeling, through dynamic regulation of gene expression networks, cell signaling pathways, and immune response mechanisms [17].

Aberrant m^6^A methylation has been associated with diabetic chronic wounds, characterized by excessive and persistent inflammatory activation, impaired neovascularization, and dysregulated ECM metabolism, thereby delaying healing [18, 19]. Additional RNA modifications also contribute to tissue repair. Beyond m^6^A, m^5^C, m^7^G, and ac^4^C exert roles in tissue-specific contexts. Notably, ac^4^C has been associated with skin repair [20], while m^5^C facilitates corneal epithelial healing [21].

Although a previous review has examined this landscape [22], the molecular mechanisms underlying RNA modifications remain poorly understood. To provide depth while maintaining clinical relevance, this review focuses on three key contexts in wound repair: cutaneous wounds resulting from burns and trauma (including diabetic wounds), fracture healing, and corneal injury. Emphasis is placed on RNA modifications—m^6^A, m^5^C, m^7^G, and ac^4^C—for which convergent mechanistic evidence is available. Evidence is explicitly qualified according to model system (*in vitro*, *in vivo* animal, and human *ex vivo*/clinical) as well as assay- and sampling-related limitations, including antibody specificity, site resolution, tissue heterogeneity, and temporal factors, to avoid overinterpretation.

## Review

### Key types of RNA modifications

#### m^6^A

**Figure 1:**
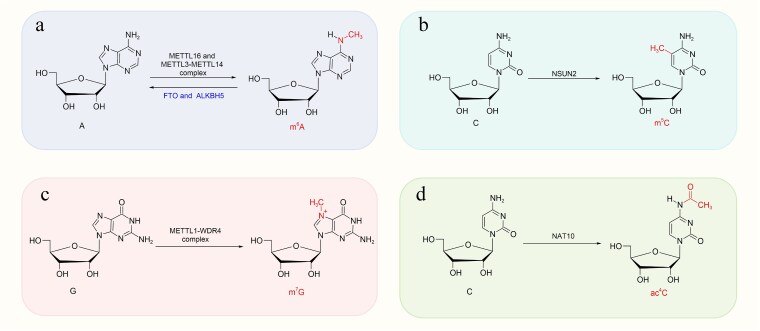
Major RNA modifications and their catalytic enzymes are implicated in wound healing. Schematic representation of key epitranscriptomic marks discussed in this review and their principal catalytic enzymes. (a) m^6^A is deposited on adenosine residues of mRNA and ncRNAs by the METTL3–METTL14 complex and METTL16, and dynamically removed by the demethylases FTO and ALKBH5. (b) m^5^C is installed on cytidine by the methyltransferase NSUN2, while the METTL1–WDR4 complex catalyzes the formation of (c) m^7^G on guanosine, and NAT10 mediates (d) ac^4^C on cytidine. These RNA modifications regulate transcript stability, splicing, nuclear export, and translation of key factors governing keratinocyte and fibroblast proliferation, inflammatory signaling, angiogenesis, osteogenic differentiation, and corneal epithelial regeneration. The coordinated actions of ‘writers’ (METTL3, METTL14, METTL16, NSUN2, METTL1, and NAT10) and ‘erasers’ (FTO and ALKBH5) establish cell-type-specific RNA modification landscapes that ultimately determine wound healing outcomes under physiological and diabetic conditions. *m*^*6*^*A N*^6^-methyladenosine, *m*^*5*^*C* 5-methylcytosine, *m*^*7*^*G N*^7^-methylguanosine, *ac*^*4*^*C N*^4^-acetylcytidine, *METTL* methyltransferase-like, *FTO* fat mass and obesity-associated protein, *ALKBH5* AlkB homolog 5, *NSUN2* NOP2/Sun RNA methyltransferase 2, *NAT10* N-acetyltransferase 10, *WDR4* WD repeat-containing protein 4. Structure created with KingDraw.

m^6^A refers to the methylation of the nitrogen atom at the sixth position of adenine in RNA (Figure 1). It is one of the most prevalent RNA modifications in mammalian cells, comprising ~0.1%–0.6% of total adenosine content in mRNA [23, 24]. m^6^A is not confined to mRNAs but is also widely distributed across diverse non-coding RNAs (ncRNAs), including ribosomal RNAs (rRNAs), transfer RNAs (tRNAs), small nuclear RNAs (snRNAs), small nucleolar RNAs (snoRNAs), microRNAs (miRNAs), long ncRNAs (lncRNAs), and circular RNAs (circRNAs). This modification predominantly occurs throughout mRNA full-length sequences and is critical for the biological functions of several ncRNAs [25–27].

The methyltransferase complex (MTC) catalyzes m^6^A modification and primarily comprises methyltransferase-like protein 3 (METTL3) and METTL14 [28, 29]. METTL3 serves as the catalytic core of the m^6^A MTC, binding S-adenosylmethionine (SAM) and catalyze RNA adenines. In contrast, METTL14, a subunit of MTC, stabilizes the complex, recognizes the RRACH RNA motif, and supports its catalytic activity [30, 31]. MTC also contains additional subunits required for localization, binding to targets (substrates), and functional regulation [32]. For instance, Wilms’ tumor 1-associated protein (WTAP) recruits METTL3 and METTL14 to form nuclear speckles [33, 34]. RNA-binding motif protein 15 directs m^6^A complexes to specific RNA sites, while VIRMA (RNA demethylase) mediates m^6^A deposition at the 3′-untranslated region (3′-UTR) and near the termination codon, regioselectively methylating purine residues within adenosine-containing RNAs [35, 36]. Furthermore, zinc finger CCCH domain-containing protein 13 anchors WTAP within nuclear speckles, thereby enhancing MTC activity [37]. METTL16 also mediates RNA methylation at U6-snRNAs, the 3′-UTRs of mRNAs, and certain lncRNAs, playing critical roles at all stages of RNA biosynthesis, processing, maturation, and post-transcriptional regulation [38].

The demethylases fat mass and obesity-associated (FTO) protein and AlkB homolog 5 (ALKBH5), members of the ALKB dioxygenase family, confer dynamic reversibility to m^6^A modifications. These enzymes require ferrous ion cofactors and α-ketoglutarate for demethylation [27, 39]. FTO, initially identified as an obesity-associated gene, also participates in other RNA modifications, including m^1^A and m^6^Am, and catalyzes m^6^A demethylation from mRNA. In contrast, ALKBH5 is essential for spermatogenesis and fertility by directly catalyzing m^6^A demethylation [40, 41]. The distinct functions of two m^6^A demethylases in various biological pathways are reflected in their differential expression across tissues and cell types.

Numerous reader proteins, including the YTH structural domain family (YTHDF1–3 and YTHDC1–2), insulin-like growth factor 2 mRNA-binding proteins (IGF2BPs), proline-rich rolled helix protein 2A, and heterogeneous nuclear ribonucleoprotein A2/B1 (HNRNPA2B1), recognize m^6^A modifications to regulate RNA fate and function [42, 43]. YTH domain-containing reader proteins, homologous to the bacterial protein YT521-B, bind directly to m^6^A modifications and function as readers [44]. IGF2BPs influence RNA stability and translation by binding to m^6^A via their KH structural domains and play critical roles in diverse biological processes [45, 46].

#### m^5^C

The m^5^C modification is a prominent RNA modification found in diverse RNA species, including mRNA, lncRNA, tRNA, rRNA, snRNA, miRNA, and enhancer RNA [47]. Its deposition is catalyzed by various methyltransferases that transfer a methyl group to the fifth carbon atom of cytosine residues; these methyltransferases vary across species [48] (Figure 1). Currently, the principal m^5^C methyltransferases identified comprise seven members of the NOL1/NOP2/SUN family (NSUN1–7) and tRNA aspartate methyltransferase 1 [49, 50].

Moreover, the TET protein family and ALKBH1 may contribute to the oxidative modification of m^5^C, potentially converting m^5^C to 5-hydroxymethylcytosine. Nevertheless, the precise demethylation activities of these proteins remain under investigation [51, 52]. Reader proteins predominantly mediate the biological functions of m^5^C modifications. In the cytoplasm, two well-characterized m^5^C reader proteins, ALYREF (RNA export factor) and Y-box binding protein 1 (YBX1), specifically recognize and bind to m^5^C modification sites, thereby regulating RNA activity [53, 54].

#### m^7^G

The m^7^G modification entails methylation of the nitrogen at the seventh position (N^7^) of guanosine in RNA (Figure 1). This modification is common in internal sequences of miRNAs, rRNAs, tRNAs, and eukaryotic mRNAs, particularly within the 5′ cap structure [55, 56].

In mammals, m^7^G modifications are predominantly catalyzed by a complex comprising Methyltransferase-like 1 (METTL1) and its cofactor WDR4 (METTL1/WDR4) [57]. METTL1 installs the m^7^G modification on target mRNAs, while WDR4 facilitates the binding of the heterodimeric complex to its targets [58]. Additionally, WBSCR22/TRMT112 MTC catalyzes m^7^G modification at position G1639 in human 18S rRNA. Functionally analogous to the Bud23/Trm112 complex in yeast, this complex installs m^7^G methylation on rRNA. WBSCR22 is named after the Williams–Beuren syndrome chromosomal region 22, with which it is associated, and TRMT112 serves as a universal activator subunit for multiple methyltransferases [59, 60]. Additionally, the 5′ cap structure of mRNAs is precisely modified by RNMT in conjunction with its cofactor RAM [61].

Although m^7^G modifications have attracted considerable attention for their roles in RNA function and disease, research on their demethylation mechanisms remains limited, and no enzymes have been conclusively identified to remove or recognize m^7^G modifications [62].

#### *N* ^4^- acetylcytidine

The ac^4^C is a conserved nucleoside modification present in eukaryotic RNAs. Although initially considered exclusive to rRNAs and tRNAs, recent studies have demonstrated that ac^4^C modification is also prevalent in the mRNAs of humans and yeast [63, 64]. The *N*-acetyltransferase-like protein 10 (NAT10) was the first enzyme identified to catalyze ac^4^C modification in eukaryotic RNAs, possessing acetyltransferase activity and RNA-binding properties. Ac^4^C denotes the acetylation of cytidine at the N4 position [65, 66].

NAT10 comprises three structural domains: an adenosine triphosphate (ATP)/guanosine triphosphate-binding domain, an ATPase domain, and an N-terminal acetylase domain. It is currently considered the only enzyme catalyzing ac^4^C modifications and functions as an ATP-dependent RNA acetyltransferase [67–69].

In human cells, nucleolar ribonucleoproteins require a box C/D snoRNA (U13) and the THUMP domain-containing protein (THUMPD1) for ac^4^C modification. These proteins interact with NAT10 to facilitate tRNA acetylation [70]. Although ac^4^C was recently identified as an RNA modification, its biological functions and mechanisms remain unclear. No demethylases or additional recognition proteins have been identified, and NAT10 remains the only documented modifying enzyme. Table 1 summarizes four RNA modifications and their associated functions [20, 21, 40, 54, 55, 57, 64, 65, 71–80].

**Table 1:** RNA modifications in wound healing: mechanisms, evidence, and limitations.

Modification	Key enzymes (writers/erasers/readers)	Primary assays used	Predominant models	Wound context	Level of evidence	Key limitations	Ref.
m^6^A	Writers: METTL3/METTL14/WTAP, KIAA1429/VIRMA, RBM15/RBM15B; Erasers: FTO/ALKBH5; Readers: YTHDC1/YTHDF1–3/IGF2BPs	MeRIP-seq; miCLIP2 (select); RIP-qPCR; site mutagenesis/rescue	HaCaT/HCEC/BMSC; mouse/rat DFUs, burn, fracture; human DFUs *ex vivo*	Skin, bone, cornea	*In vitro* + *in vivo* animal; limited human *ex vivo*	Antibody specificity; limited site-level validation; species/phase differences	[40, 71–73]
m^5^C	Writers: NSUN1–7/DNMT2 (TRDMT1); Erasers: TET1–3; Readers: Aly/REF, YBX1 (putative)	Bisulfite RNA-seq; RNA-IP; functional rescue	HCEC; corneal injury models	Cornea	*In vitro* + *in vivo* animal	Bisulfite artifacts; reader specificity	[21, 54, 74]
m^7^G	Writers: METTL1/WDR4 (internal/tRNA); Readers: EIF4E, NCBP1/NCBP2 (CBP80/CBP20), AGO2, LARP1, DCP2	mRNA/tRNA mapping; LC–MS; ribosome profiling	MSCs; fracture/bone models	Bone/angiogenesis	*In vitro* + *in vivo* animal	Distinguishing internal *vs* cap m^7^G	[55, 57, 75, 76]
ac^4^C	Writers: NAT10	ac^4^C-seq; LC–MS; reporter assays	Keratinocytes; skin injury	Skin, bone	*In vitro* + *in vivo* animal	Limited site-level resolution	[20, 64, 65, 77–80]

*METTL3* methyltransferase-like 3, *METTL14* methyltransferase-like 14, *WTAP* Wilms tumor 1-associated protein, *RBM15* RNA binding motif protein 15, *RBM15B* RNA binding motif protein 15B, *FTO* fat mass and obesity-associated protein, *ALKBH5* ALKB homolog 5, *YTHDC1* YTH domain-containing 1, *YTHDF1–3* YTH N^6^-methyladenosine RNA binding protein F1–3, *IGF2BPs* insulin-like growth factor 2 mRNA-binding proteins, *NSUN1–7* NOP2/Sun RNA methyltransferase family members 1–7, *TET1–3* ten-eleven translocation methylcytosine dioxygenase 1–3, *YBX1* Y-box binding protein 1, *WDR4* WD repeat domain 4, *EIF4E* eukaryotic translation initiation factor 4E, *AGO2* Argonaute 2, *LARP1* La-related protein 1, *DCP2* mRNA decapping enzyme 2, *NAT10* N-acetyltransferase 10, *HaCaT* human keratinocyte cell line, *HCEC* human corneal endothelial cell, *BMSC* bone marrow stromal cell, *MSC* mesenchymal stem cell

### The role of *N^6^*-methyladenosine modifications in skin wound healing

#### Skin structure, function, and the wound repair paradigm

The skin is a continuously self-renewing organ system that covers the body surface, forming a dynamic interface between the organism and the external environment as well as providing protection against physical, chemical, and biological agents [81]. Beyond serving as the primary barrier, the skin functions as an active sensory and regulatory center [82]. Cutaneous nerve endings detect touch, pressure, temperature, and pain, enabling rapid responses to environmental changes [83]. The skin also contributes to thermoregulation through sweat secretion and modulation of subcutaneous blood vessel dilation or constriction. Structurally, the skin comprises three layers: the epidermis, dermis, and subcutaneous tissue. The epidermis undergoes continuous exfoliation and renewal; the dermis confers elasticity and mechanical strength; and the subcutaneous tissue cushions underlying structures and serves as an energy reservoir. This organized, multilayered architecture underpins the skin’s complex functional capabilities [84].

Wound healing is a complex, multistage process that requires coordinated interactions among various tissues and cell lines [12]. Cutaneous wound repair involves a highly regulated sequence of overlapping events, including cell migration and proliferation, ECM deposition and remodeling, inflammatory responses, angiogenesis, and additional cellular and molecular processes, such as gene expression regulation mediated by m^6^A RNA methylation [14, 85].

In the skin, RNA modifications regulate three key modules with phase-coupled dynamics: (i) inflammation and macrophage polarization; (ii) keratinocyte and fibroblast proliferation with metabolic reprogramming; (iii) (lymph)angiogenesis and ECM remodeling. Rather than presenting study findings chronologically, this review summarizes according to these functional modules.

#### Diabetic skin wounds

##### Pathophysiology, clinical burden, and microenvironmental drivers

In diabetes, cutaneous repair is typically delayed, characterized by prolonged inflammation and impaired epithelial regeneration [86]. Diabetic foot ulcers (DFUs) are among the most severe complications, frequently leading to lower limb amputation and increased mortality, affecting ~15% of individuals with type 2 diabetes [87]. DFUs often arise from hyperglycemia, which causes various systemic complications and induces pathological alterations in the local wound microenvironment. These changes include persistent inflammatory responses, microcirculatory dysfunction, elevated oxidative stress, neurological impairments, and accumulation of advanced glycation end products (AGEs) [88, 89].

Sustained hyperglycemia further impairs healing through disruption of keratinocyte behavior, vascular function, and immune responses. Hyperglycemia significantly inhibits keratinocyte migration by blocking the autophagy signaling pathway involving p38/MAPK, thereby delaying re-epithelialization [90, 91]. Furthermore, hyperglycemia dynamically regulates cellular function through epigenetics, particularly m^6^A RNA methylation. Recent studies have indicated that m^6^A modification modulates autophagy, inflammation, and metabolism, highlighting its central role in diabetic wound healing. Key regulators include METTL3, IGF2BP2, FTO, and ALKBH5 [16, 92].

##### Writers (METTL3/METTL14) in diabetic wound healing

AGEs alter the m^6^A modification profile in human dermal fibroblasts by increasing METTL14 expression and elevating m^6^A levels, resulting in fibroblast dysfunction and impairing wound healing [93]. Notably, METTL14 enhances suppressor of cytokine signaling 1 (Socs1) expression via YTHDF1-mediated m^6^A methylation, promoting macrophage M2 polarization, angiogenesis, and fibroblast activity, thereby facilitating skin wound healing [94]. Similarly, METTL3 positively regulates wound healing by upregulating the m^6^A/HNRNPA2B1/DNMT1 signaling pathway in keratinocytes. Moreover, elevated lactate in the wound microenvironment modulates METTL3 expression through histone H3K18 lactylation [95].

**Figure 2:**
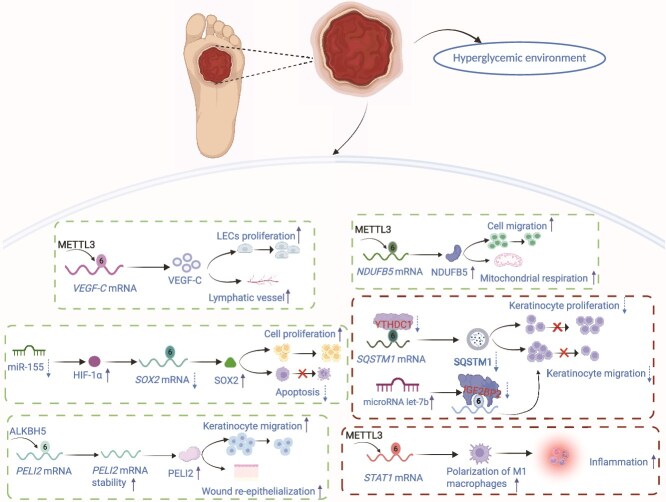
m^6^A-mediated epitranscriptomic regulation in diabetic wound healing. Schematic depicting cell-type-specific roles of m^6^A machinery in hyperglycemic wound environments. Upper pathway: METTL3-dependent m^6^A modification stabilizes *VEGF-C* mRNA in LECs, promoting lymphangiogenesis and edema resolution. Middle pathway: in keratinocytes, YTHDC1 mediates nuclear export of m^6^A-modified transcripts, including miR-155 precursors targeting HIF-1α, while stabilizing *SOX2* and *SQSTM1* mRNAs to enhance proliferation and inhibit apoptosis. Lower pathway: ALKBH5-mediated demethylation stabilizes *PELI2* mRNA, facilitating keratinocyte migration, whereas METTL3 methylates STAT1 transcripts in macrophages, promoting M1 polarization and sustaining inflammation—a hallmark of diabetic wound pathology. IGF2BP2 competes with let-7b miRNA for binding, thereby protecting target mRNAs from degradation and facilitating re-epithelialization. Solid arrows indicate activation, and dashed lines represent inhibition. Green boxes denote pro-healing mechanisms, and red boxes indicate pathological processes exacerbated in diabetes. The interplay between these pathways governs wound closure kinetics and scar quality. Therapeutic modulation of m*^6^*A writers and erasers represents a promising strategy for diabetic wound management. *METTL3* methyltransferase-like 3, *ALKBH5* AlkB homolog 5, *YTHDC1* YTH domain-containing protein 1, *VEGF-C* vascular endothelial growth factor C, *LECs* lymphatic endothelial cells, *SQSTM1* sequestosome 1, *SOX2* SRY-box transcription factor 2, *HIF-1α* hypoxia-inducible factor 1α, *IGF2BP2* insulin-like growth factor 2 mRNA-binding protein 2, *PELI2* pellino E3 ubiquitin protein ligase 2, *STAT1* signal transducer and activator of transcription 1. Figure created with BioRender.com (licenses: LS296U0EHZ).

Transient hyperglycemia reduces RNA methylation levels, impairs cellular metabolism, and delays wound healing by decreasing METTL3 expression in vascular endothelial cells. Conversely, Nocardia erythropolis cell wall skeleton stabilizes mitochondrial function by upregulating METTL3, which enhances m^6^A modification of *Cds2* mRNA, boosts ATP production, and promotes angiogenesis, ultimately accelerating wound healing [96]. Furthermore, Liu *et al.* [97] demonstrated that the m^6^A methyltransferase METTL3 promotes M1 macrophage polarization via signal transducer and activator of transcription 1 (*STAT1*) mRNA methylation, contributing to chronic inflammation in diabetic wounds. METTL3 also upregulates NADH:ubiquinone oxidoreductase subunit B5 (NDUFB5) expression via m^6^A modifications, thereby enhancing cell migration and mitochondrial respiration, and improving healing in DFUs (Figure 2) [71].

##### Erasers (FTO/ALKBH5) and context-dependent effects

In DFUs, elevated FTO protein expression associates with reduced m^6^A RNA methylation levels, dysregulating glucose metabolism-related genes, including *FOXO1*, *G6PC*, and *DGAT2*, and consequently exacerbating impaired wound healing [98]. Furthermore, Zheng *et al.* [99] developed a nanocolloidal hydrogel capable of scavenging reactive oxygen species (ROS) and delivering FTO inhibitors. This strategy markedly improved wound healing and epidermal regeneration in diabetic rats and mouse models by upregulating *MMP9* mRNA levels.

In addition, FTO upregulates TRIB3 expression by demethylating its mRNA and preventing YTHDF2-mediated degradation, thereby promoting autophagy and accelerating diabetic skin wound healing [100]. However, the role of FTO in wound healing remains controversial in the scientific literature. Some studies have associated elevated FTO expression with impaired healing [98], whereas others indicate that it may promote healing through distinct molecular pathways. The m^6^A regulatory system is highly context-dependent; therefore, FTO function varies according to cell type, disease stage, and target genes. Consequently, categorizing its effects as uniformly ‘beneficial’ or ‘detrimental’ is misleading.

Remethylation of the m^6^A demethylation is predominantly regulated by demethylases, specifically FTO and ALKBH5. In patients with diabetes, FTO expression is elevated in response to reduced m^6^A levels [101, 102]. FTO modulates macrophage polarization through NF-κB and STAT1 signaling pathways during cutaneous inflammation and wound healing [103]. Conversely, ALKBH5 expression is significantly increased in the wound margin epidermis, and its absence impairs keratinocyte migration and healing. The downstream target of ALKBH5, pellino E3 ubiquitin protein ligase 2 (PELI2), exhibits enhanced mRNA stability through m^6^A demethylation, thereby promoting keratinocyte migration and wound re-epithelialization (Figure 2) [16].

##### *N^6^*-methyladenosine and binding proteins: YTHDC1, YTHDF1, IGF2BP2

Modulation of m^6^A modifications dynamically alters cellular functions by regulating autophagy-related pathways and global gene expression. The YTHDC1 protein modulates sequestosome 1 (SQSTM1) levels, which in turn influence autophagic flux. A substantial reduction in SQSTM1 can inhibit autophagy, impair keratinocyte migration and proliferation, and delay wound healing (Figure 2) [104].

m^6^A modifications regulate gene expression and influence cellular functions. m^6^A facilitates wound healing by enhancing fibroblast and keratinocyte proliferation and migration through modulation of exosomal proteins and ncRNAs, including circRNAs. SPARC plays a key role in wound healing by regulating fibroblast migration, a critical process for large wound healing, and its absence substantially impairs healing [105]. Additionally, Circ-Amotl1 binds to IGF2BP2 within exosomes, induces SPARC expression, and accelerates healing by enhancing fibroblast proliferation and migration [106]. Vascular endothelial growth factor C (VEGF-C) is a major mediator of angiogenesis and lymphangiogenesis during wound healing [107]. Adipose-derived mesenchymal stem cells (ADSCs) regulate VEGF-C expression via the m^6^A modification pathway, specifically through METTL3, thereby promoting lymphatic endothelial cell (LEC) proliferation and lymphangiogenesis. Overall, these mechanisms accelerate the healing of DFUs (Figure 2).

This regulatory mechanism depends on post-transcriptional regulation of VEGF-C via the METTL3/IGF2BP2-m^6^A signaling pathway [108]. Concurrently, IGF2BP2 within the diabetic microenvironment promotes keratinocyte proliferation and migration by stabilizing heparanase [109]. Conversely, miRNA let-7b inhibits IGF2BP2 activity, reducing keratinocyte migration and impairing wound healing under diabetic conditions [110].

##### Autophagy regulation via *N^6^*-methyladenosine modifications

*In vitro* and *ex vivo* studies indicate that AGEs activate the FOXO1 signaling pathway to stimulate autophagy. However, the overall impact on wound healing probably depends on the dose, duration of exposure, and cellular state. Excessive autophagy can disrupt cell function or induce cell death, thereby impairing the healing process [111]. Additionally, lncCCKAR5 regulates autophagy through m^6^A modification [102].

##### Integrated signaling: inflammation, metabolism, and stem cell maintenance

Dysregulated inflammatory responses impair wound healing in diabetes. Interleukin (IL)-6 compromises keratinocyte viability, glycolysis, and inflammatory regulation through METTL14-mediated m^6^A modifications, thereby hindering wound healing [112]. Additionally, m^6^A-modified Pvt1 contributes to skin wound repair by stabilizing MYC protein, which regulates epidermal stem cell properties and maintains epidermal tissue homeostasis [113]. Inhibition of miR-155 upregulates hypoxia-inducible factor 1-alpha (HIF-1α) expression, which reduces m^6^A modification of SRY-box transcription factor 2 (*SOX2*) mRNA, induces SOX2 expression, and activates EGFR/MEK/ERK signaling pathway. This cascade enhances cell proliferation and migration while modulating blood flow to support tissue regeneration. Conversely, HIF-1α knockdown reverses these effects (Figure 2) [114].

In summary, m^6^A RNA methylation plays a central role in the molecular network of diabetic wound healing by precisely regulating mechanisms, such as autophagy, inflammatory responses, and cellular functions, providing a robust theoretical foundation and promising avenues for the reparative treatment of refractory wounds.

#### Burned skin wounds

##### Clinical burden, structural damage, and infection risk

According to the World Health Organization statistics, ~11 million individuals worldwide sustain burns of varying severity annually, resulting in nearly 180 000 deaths from burns and their complications [115]. As the largest organ of the body, the skin provides essential barrier, sensory, and immunological functions. Burn injury represents one of the most common and devastating forms of skin trauma [116]. Human skin consists of two primary anatomical layers: the epidermis, which forms the outer barrier in contact with the external environment, and the dermis, which contains blood vessels, nerves, and appendages, such as hair follicles, sweat glands, and sebaceous glands [117]. The dermis confers mechanical resilience by resisting deformations from external stimuli. Burns can destroy skin appendages and the epidermal-dermal architecture, resulting in scarring, pain, and infection [118]. In severe cases, burn injuries can extensively compromise the entire skin structure, including adjacent subcutaneous tissues, blood vessels, and nerves, thereby substantially impairing skin function [119]. Moreover, disruption of the skin barrier in eschar regions allows pathogen entry, including bacteria, and markedly increases the risk of infection [120].

##### *N^6^-*methyladenosine dynamics, FTO/METTL14 function, and scar formation

Burn injuries not only cause structural damage but also induce significant alterations in gene expression and epigenetic regulation. Among these, RNA methylation modifications, particularly m^6^A, constitute a crucial post-transcriptional regulatory mechanism that plays a vital role in post-burn repair [121]. Evidence indicates that m^6^A modifications dynamically regulate methylation levels to modulate keratinocyte proliferation, migration, differentiation, and ECM remodeling [122, 123], all of which are essential for burn wound healing and tissue regeneration.

**Figure 3:**
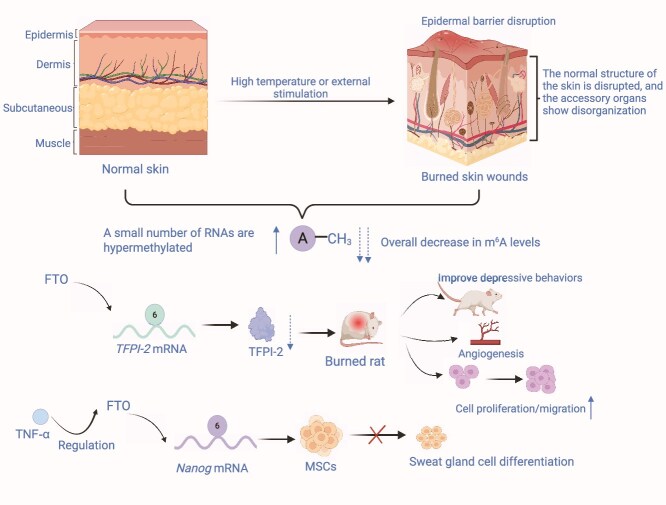
m^6^A regulatory dynamics in burn-induced skin injury and repair. Thermal injury disrupts epidermal barrier integrity and induces global alterations in the m^6^A epitranscriptome, including an overall reduction in m^6^A methylation levels and selective hypermethylation of specific transcripts. The m^6^A demethylase FTO serves as a central regulator in burn wound repair. FTO-mediated demethylation stabilizes *TFPI-2* mRNA, promoting angiogenesis, keratinocyte proliferation, and migration, and accelerating wound re-epithelialization. In MSCs, FTO regulates m^6^A modification of *Nanog* mRNA, enhancing stem cell pluripotency and facilitating sweat gland cell differentiation, which is a critical process for restoring skin appendage function following severe burns. FTO also modulates the expression of inflammatory mediators, including TNF-α, thereby attenuating burn-induced systemic inflammation and ameliorating depressive behaviors observed in burn injury models. Collectively, these findings highlight FTO as a key epitranscriptomic regulator that integrates vascular, stromal, and neuroimmune signals via m^6^A demethylation to modulate stem cell fate and promote tissue regeneration after thermal injury. *TFPI-2* tissue factor pathway inhibitor 2, *FTO* fat mass and obesity-associated protein, *MSCs* mesenchymal stem cells, *TNF-α* tumor necrosis factor-alpha. Figure created with BioRender.com (licenses: AC296U0T67).

Following burn injury, m^6^A levels in skin tissues undergo significant alterations, resulting in hypomethylation of numerous mRNAs and lncRNAs, whereas only a subset of RNAs exhibit hypermethylation. Reduced expression of hypomethylated mRNAs impairs multiple pathways involved in wound healing [122]. Another study indicates that FTO proteins play a critical role in wound healing by modulating m^6^A methylation levels and regulating tissue factor pathway inhibitor-2 (TFPI-2) expression. TFPI-2 overexpression inhibits FTO-mediated promotion of keratinocyte proliferation, migration, and angiogenesis. In a burn rat model, FTO overexpression accelerated wound healing and ameliorated burn-induced depression-like behavior (Figure 3) [123].

Notably, these findings were derived from rat models, and differences may exist in skin development, regulatory mechanisms, and wound repair timing between rats and humans. Burn datasets are limited in duration and rely on phase-resolved sampling. Moreover, antibody-based mapping of m^6^A requires site-specific validation. To enhance translational relevance, molecular data should ideally be correlated with behavioral outcomes, such as pain assessment, and with scarring endpoints.

In addition to FTO, tumor necrosis factor-alpha (TNF-α) modulates *Nanog* mRNA stability by regulating FTO-mediated m^6^A demethylation, thereby inhibiting the differentiation of mesenchymal stem cells (MSCs) into sweat gland cells [124]. METTL14 also regulates the translation of Col17a1, Itgβ4, and Itgα6 via m^6^A modifications, thereby maintaining epidermal homeostasis. The absence of METTL14 depletes stem cells, thickens the epidermis, and reduces the expression of these critical proteins, impeding cellular self-renewal [17]. Analyses of hyperplastic scar tissue using m^6^A and RNA sequencing reveal a distinct m^6^A modification pattern, with modifier genes closely associated with fibrosis-related pathways [125].

In summary, dynamic regulation of key gene expression by m^6^A modification constitutes a central mechanism in burn wound repair. FTO inhibitors suppress excessive inflammatory responses mediated via m^6^A, while METTL14 activation inhibits the expression of scar-related proteins. Together, these factors offer a dual approach to addressing the clinical challenge of dysregulated inflammation and scar formation, providing a novel avenue for personalized burn therapy.

#### Other skin wound healing

Burn injuries are not the only context in which m^6^A modification plays a critical regulatory role. This epitranscriptomic modification also contributes to repair processes in ultraviolet B (UVB)-induced sunburn and contact thermal-induced scald injuries. Sunburn, primarily caused by midwave ultraviolet radiation, is pathologically characterized by DNA damage, cell death, and inflammation [126]. Scald injuries result from exposure to hot liquids or solid surfaces, triggering inflammatory activation and tissue necrosis during the acute phase [127]. In contact thermal injury from hot metals, skin RNA expression profiles are similarly altered, with rapid activation of inflammatory responses and increased production of pro-inflammatory cytokines, including IL-1β and TNF-α [128].

Studies indicate that in various types of thermal damage, m^6^A-related modifying enzymes distinctly regulate damage and repair processes. In UVB-irradiated HaCaT cells, ALKBH5 expression is significantly elevated, thereby promoting apoptosis, increasing IL-1β, IL-18, and TNF-α levels, and upregulating pyroptosis-related proteins, including GSDMD, Caspase-1, and Caspase-4. ALKBH5 inhibition effectively alleviates pyroptosis and inflammatory responses, thereby reducing UVB-induced skin damage in mice [129]. In contrast, during thermal burn repair, METTL3 mediates m^6^A modification of *CCNB1* mRNA via ASC-modified exosomes, significantly enhancing the migration and proliferation of skin fibroblasts and providing a novel therapeutic strategy for burn repair [130].

As a key post-transcriptional regulatory mechanism, m^6^A RNA methylation plays a critical role in repairing various skin injuries, including diabetic wounds, burns, ultraviolet-induced damage, and scalds. Despite variations in etiology and microenvironment among wound types, m^6^A modifications dynamically and reversibly regulate the inflammatory, proliferative, and remodeling phases of wound healing. The principal regulatory mechanisms of m^6^A at each stage of skin wound healing are systematically summarized in Table 2 [16, 17, 71, 73, 97, 100, 104, 108, 123, 131–139].

**Table 2:** Key functions of m^6^A regulators in wound healing stages.

Wound type	Phase	Regulator (writer/eraser/reader)	Target/pathway	Functional outcome	Model system	Evidence type	Strength of evidence	Reference
Diabetic skin	Inflammation	METTL3	*STAT1* mRNA	↑M1 polarization, chronic inflammation	Mouse *db*/*db*, human DFU biopsies	*In vivo* + *ex vivo*	⊕ ⊕ ⊕⊝(Moderate)	[97]
	Proliferation	ALKBH5	*PELI2* mRNA	↑Keratinocyte migration, re-epithelialization	Mouse *db*/*db*, human keratinocytes	*In vivo* + *in vitro*	⊕ ⊕ ⊕ ⊕ (High)	[16]
	Proliferation	METTL3	*NDUFB5* mRNA	↑Mitochondrial respiration, cell migration	Mouse STZ-DM, primary keratinocytes	*In vivo* + *in vitro*	⊕ ⊕ ⊕⊝ (Moderate)	[71]
	Proliferation	YTHDC1	*SQSTM1* mRNA	Autophagy regulation, keratinocyte function	Mouse *db*/*db*, human keratinocytes	*In vivo* + *in vitro*	⊕ ⊕ ⊕⊝ (Moderate)	[104]
	Angiogenesis	METTL3/IGF2BP2	*VEGF-C* mRNA	↑Lymphangiogenesis, wound closure	Mouse *db*/*db*, ADSC exosomes	*In vivo*	⊕ ⊕ ⊕⊝ (Moderate)	[108]
	Remodeling	FTO	*TRIB3* mRNA	↑Autophagy, accelerated healing	Mouse *db*/*db*	*In vivo*	⊕ ⊕ ⊝⊝ (Low–Moderate)	[100]
Burn	Proliferation	FTO	*TFPI-2* mRNA	↑Keratinocyte proliferation, angiogenesis	Rat burn model	*In vivo*	⊕ ⊕ ⊝⊝ (Low–Moderate)	[123]
	Remodeling	METTL14	Col17a1, Itgβ4, Itgα6	Epidermal homeostasis, stem cell maintenance	Mouse knockout model	*In vivo*	⊕ ⊕ ⊕⊝ (Moderate)	[17]
Fracture	Proliferation	METTL3	Pth1r, LINC00657/miR-144-3p/BMPR1B	↑Osteogenic differentiation	Mouse BMSC, fracture models	*In vivo* + *in vitro*	⊕ ⊕ ⊕⊝ (Moderate)	[131, 132]
	Proliferation	YTHDF1	ZNF839, THBS1	↑Translation of osteogenic factors under hypoxia	Mouse BMSC, fracture callus	*In vivo* + *in vitro*	⊕ ⊕ ⊕⊝ (Moderate)	[133–135]
	Remodeling	FTO	*PPARG* mRNA	↓Adipogenesis, ↑osteogenesis	Mouse BMSC	*In vivo* + *in vitro*	⊕ ⊕ ⊕⊝ (Moderate)	[136]
Cornea	Proliferation	METTL3	AHNAK, DDIT4	↑Corneal limbal stem cell proliferation/migration	Mouse alkali burn	*In vivo*	⊕ ⊕ ⊕⊝ (Moderate)	[73]
	Angiogenesis	WTAP	*HIF-1α* mRNA	↑Macrophage VEGFA/C/D secretion, CNV	Mouse CNV model	*In vivo*	⊕ ⊕ ⊕⊝ (Moderate)	[137]
	Proliferation	YTHDF3	*THBS2* mRNA	↑Epithelial healing via Wnt/β-catenin in diabetes	Diabetic mouse cornea	*In vivo*	⊕ ⊕ ⊕⊝ (Moderate)	[138]
	Fibrosis	METTL3	COL3A1, α-SMA, fibronectin	↓Fibrosis markers when silenced	Mouse alkali burn, TGF-β1-treated cells	*In vivo* + *in vitro*	⊕ ⊕ ⊝⊝ (Low–Moderate)	[139]

*METTL3* methyltransferase-like 3, *METTL14* methyltransferase-like 14, *FTO* fat mass and obesity-associated protein, *ALKBH5* ALKB homolog 5, *IGF2BP2* insulin-like growth factor 2 mRNA-binding protein 2, *YTHDC1* YTH domain-containing 1, *STAT1* signal transducer and activator of transcription 1, *DFU* diabetic foot ulcer, *PELI2* Pellino E3 ubiquitin protein ligase family member 2, *NDUFB5* NADH:ubiquinone oxidoreductase subunit B5, *SQSTM1* sequestosome 1, *VEGF-C* vascular endothelial growth factor C, *ADSC* adipose-derived stem cell, *TRIB3* Tribbles pseudokinase 3, *TFPI-2* tissue factor pathway inhibitor 2, *BMPR1B* Bone morphogenetic protein receptor type 1B, *THBS1* thrombospondin 1, *HIF-1α* hypoxia-inducible factor 1-alpha, *THBS2* thrombospondin 2, *α-SMA* alpha-smooth muscle actin, *TGF-β1* transforming growth factor beta 1.

Evidence grading criteria:

• ⊕ ⊕ ⊕ ⊕ (High): Multiple independent *in vivo* studies + mechanistic validation + partial human validation (*ex vivo* tissue or biopsy-based analysis).

• ⊕ ⊕ ⊕⊝ (Moderate): ≥1 *in vivo* rodent study + robust *in vitro* mechanism; lacks human validation.

• ⊕ ⊕ ⊝⊝ (Low–Moderate): Single *in vivo* study or predominantly *in vitro* data; site-resolution or antibody validation absent.

• ⊕⊝⊝⊝ (Very Low): *In vitro* only; preliminary correlation; requires independent replication.

### Evidence appraisal, knowledge gaps, and translational relevance in skin

Strength of Evidence: Multiple *in vivo* rodent models of diabetes and burn injury, along with *ex vivo* studies of human DFUs and skin, support roles for METTL3, ALKBH5, FTO, YTHDC1, and IGF2BP2 [16, 103, 109]. Nonetheless, data on human clinical intervention remain limited.

Convergence: METTL3 enhances keratinocyte migration and mitochondrial respiration during the proliferative phase via NDUFB5 [71]. YTHDC1 regulates autophagy and keratinocyte migration from the inflammatory to the proliferative phases through SQSTM1 [104]. Additionally, the METTL3/IGF2BP2 axis promotes lymphangiogenesis during proliferation via VEGF-C [108].

Divergence/Contradictions: Although FTO expression is elevated in DFUs and associates with impaired healing [98], its activity can enhance keratinocyte proliferation and angiogenesis in burn injuries through TFPI-2 demethylation [123]. These discrepancies potentially reflect differences in timing and tissue context.

Gaps: (i) Phase- and cell-type-specific maps of modification in human DFUs and burn margins; (ii) causal, site-specific editing of key transcripts, including SOX2, PELI2, and VEGF-C; (iii) validation of macrophage-targeted epitranscriptomic interventions aimed at restoring M1/M2 balance.

Limitations and Model Considerations: Many DFUs studies employ keratinocyte and fibroblast cell lines and rodent wound models, which differ substantially from human skin architecture, including epidermal thickness, junctional organization, and adnexal density [140, 141]. Bulk profiling approaches obscure the contributions of cell types undergoing phase transitions. Improved causal inference and target prioritization will benefit from the application of single-cell and spatial methodologies, as well as *ex vivo* analyses of human DFUs and burn margins.

Translational Relevance: Topical hydrogels, including ROS-scavenging FTO inhibitors, and exosome-based therapies, such as METTL3-modified ASC exosomes, have demonstrated efficacy in rodents [99, 130], supporting the feasibility of phase-specific, localized delivery for DFUs and burn wounds.

### The role of *N^6^*-methyladenosine modifications in fracture healing

#### Clinical burden and the fracture healing cascade

Fractures are a major global health concern, causing substantial reductions in quality of life and increased physical disability. Among traumatic injuries involving major organs, fracture is the most common type of injury in humans [142, 143]. A comprehensive understanding of the mechanisms underlying fracture occurrence and healing is essential, given the significant impact of these injuries on patient outcomes. Fractures typically result from external traumatic events; however, intrinsic risk factors, such as osteoporosis and decreased bone mineral density, markedly increase fracture susceptibility [144]. Populations at elevated risk include manual laborers, individuals with diabetes, athletes, and older adults [145]. Fracture healing is a complex, dynamic process that progresses through sequential phases: hematoma formation, cartilaginous callus development, bone tissue formation, and final remodeling of the healed tissue [146].

#### Core *N^6^-*methyladenosine writers (METTL3/METTL14) in osteogenesis and repair

Beyond mechanical influences, m^6^A RNA methylation, a key post-transcriptional regulatory mechanism, plays a critical role in fracture repair. It regulates osteogenic differentiation, osteoclast activity, and bone microenvironment homeostasis [147, 148]. The m^6^A methyltransferases METTL3 and METTL14 regulate osteoblast differentiation, thereby influencing bone formation. Specifically, METTL3 enhances MYD88 expression through m^6^A modification, thereby activating NF-κB signaling and inhibiting osteogenic differentiation [149].

**Figure 4:**
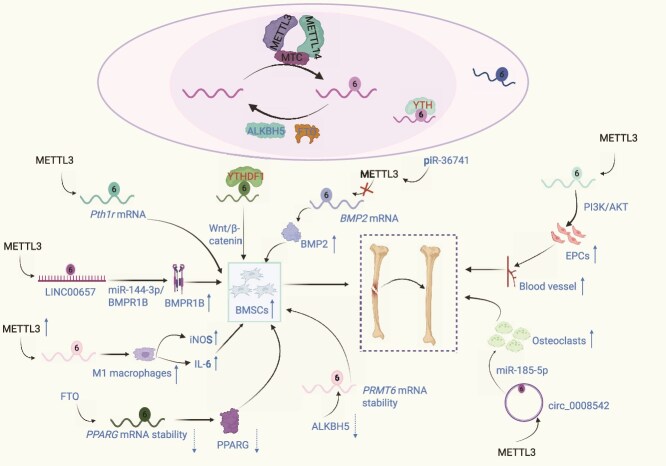
Role of m^6^A modifications in fracture healing and osteogenesis. Fracture healing involves coordinated osteogenesis, angiogenesis, and immune regulation, each of which is governed by cell-type-specific m^6^A machinery. In BMSCs, METTL3 enhances m^6^A methylation of *BMP2* mRNA to activate Wnt/β-catenin signaling and promote osteoblast differentiation, while stabilizing *Pth1r* mRNA via YTHDF1-mediated translation, thereby further driving osteogenic commitment through the activation of PI3K/AKT pathway. In EPCs, METTL3 upregulates BMP2 to stimulate angiogenesis, coupling vascular invasion with bone formation. Conversely, ALKBH5 demethylates *PPARG* mRNA in BMSCs, destabilizes anti-osteogenic transcripts, and facilitates mineralization. Within the inflammatory compartment, METTL3 promotes M1 macrophage polarization by stabilizing iNOS and IL-6 transcripts, thereby sustaining early inflammatory responses. The lncRNA LINC00657 acts as a molecular scaffold, sequestering miR-144-3p to de-repress BMPR1B and promote BMP signaling. Meanwhile, FTO stabilizes *PRMT6* mRNA by antagonizing miR-185-5p, thereby enhancing epigenetic regulation of osteogenesis. The circRNA circ_0008542 modulates METTL3 activity, fine-tuning the balance between inflammation and regeneration. Collectively, these m^6^A-dependent regulatory networks integrate stem cell differentiation, angiogenesis, and immune modulation to orchestrate successful bone healing. *BMSCs* bone marrow mesenchymal stem cells, *EPCs* endothelial progenitor cells, *METTL3* methyltransferase-like 3, *ALKBH5* ALKB homolog 5, *FTO* fat mass and obesity-associated protein, *Pth1r* parathyroid hormone 1 receptor, *iNOS* inducible nitric oxide synthase, *IL-6* interleukin-6. Figure created with BioRender.com (licenses: QN296U18AO).

Moreover, METTL3 modifies *Pth1r* mRNA via m^6^A methylation, thereby enhancing MSC osteogenic differentiation [131]. Subsequent studies revealed that METTL3 regulates the osteogenic differentiation of bone marrow MSCs (BMSCs). METTL3 methylates LINC00657 at m^6^A sites, enabling it to act as a competitive endogenous RNA with miR-144-3p, which upregulates BMPR1B and promotes osteogenic differentiation of BMSCs [132]. Additional evidence indicates that piR-36 741 enhances BMP2 expression and enhances osteogenic differentiation by inhibiting METTL3-mediated m^6^A modification of *BMP2* mRNA (Figure 4) [150]. Transcriptome-wide mapping techniques based on enrichment, such as MeRIP-seq or m^6^A-seq, provide broad coverage but low site resolution and limited antibody specificity [151]. To identify sentinel sites and assess occupancy changes, site-resolved approaches, including miCLIP2 for m^6^A, deamination adjacent to RNA modification target sequencing (DART-seq), and MAZTER-seq for m^6^A-sensitive sites, are recommended. Furthermore, to enhance the reliability and specificity of the results, orthogonal validation strategies, including RNA immunoprecipitation followed by quantitative polymerase chain reaction (RIP-qPCR) with independent antibodies and methylation-deficient mutants, should be employed [152, 153].

On the other hand, m^6^A methylation of circ-0008542 promotes osteoclast differentiation and bone resorption by binding METTL3 and sponging miR-185-5p [154]. Additionally, MHL, an extract derived from the Chinese herb *Drynaria*, enhances BMSC osteogenic differentiation by upregulating METTL3 and METTL14, increasing alkaline phosphatase (ALP) activity and osteogenic marker expression, including *Osterix* and *Osteocalcin* [155].

METTL3 enhances the angiogenic potential of endothelial progenitor cells (EPCs) via the PI3K/AKT signaling pathway, contributing to bone regeneration and angiogenesis (Figure 4) [156] Consequently, downregulation of METTL3 in BMSCs impairs osteogenic differentiation and bone mass gain, whereas its overexpression significantly enhances both processes [157]. Furthermore, METTL3 regulates macrophage polarization, influencing bone repair. METTL3 expression is elevated in pro-inflammatory macrophages (M1), and its overexpression stimulates M1 macrophages to secrete IL-6 and inducible nitric oxide synthase (iNOS). Additionally, it stimulates BMSC migration and osteogenesis [158].

METTL3 promotes osteogenic differentiation by inhibiting the maturation of miR-7212-5p, which targets FGFR3 to suppress osteoblast differentiation. The METTL3/miR-7212-5p/FGFR3 axis is essential for fracture healing [147]. Consistently, METTL3 expression increases during osteogenic differentiation, and its knockdown reduces osteogenic marker expression and disrupts the SMAD signaling pathway [159, 160]. In osteoclasts, METTL3 knockdown enlarges cell size but diminishes bone resorption capacity [161]. METTL14 also plays a pivotal role in regulating osteogenesis. Hydrogen peroxide treatment suppresses osteogenic differentiation and glycolysis, whereas METTL14 overexpression reverses these effects [162]. Furthermore, METTL14-mediated m^6^A modification of Beclin-1 enhances autophagy and osteogenesis [163].

#### Core *N^6^*-methyladenosine erasers (FTO/ALKBH5) in lineage allocation

FTO enhances osteogenic differentiation of MSCs by regulating *PPARG* mRNA stability. It suppresses *PPARG* expression and decreases its mRNA stability through a YTHDF1-dependent mechanism (Figure 4). PPARG inhibition upregulates osteogenic markers, such as ALPL and OPN, whereas PPARG overexpression inhibits osteogenesis. These findings demonstrate that FTO enhances osteogenic differentiation by post-transcriptionally suppressing PPARG [136]. Exosome-delivered miR-22-3p promotes osteogenic differentiation of BMSCs by downregulating FTO and inhibiting the MYC/PI3K/AKT signaling pathway [164]. Under AGE-induced damage, FTO inhibition restores the osteogenic capacity of BMSCs by accelerating *SOST* mRNA degradation and activating the Wnt signaling pathway [165].

In addition, modulation of FTO-mediated m^6^A modifications via toll-like receptor 4 inhibition prevents bone loss [166]. By targeting the FTO gene, miR-149-3p overexpression promotes osteogenic differentiation while inhibiting lipogenic differentiation in BMSCs [167]. In contrast, ALKBH5, another m^6^A demethylase, acts as a negative regulator of osteoblast differentiation by modulating *PRMT6* mRNA stability through m^6^A demethylation, thereby influencing MSC osteogenic differentiation. ALKBH5 knockdown in BMSCs increases bone mass in rodent models, further confirming its inhibitory role in MSC osteogenic differentiation [72].

#### The role of *N^6^*-methyladenosine readers and binding proteins

Fracture healing is significantly influenced by the IGFBP3/miR-23a-3p/SMAD5 axis. IGFBP3 substantially increases miR-23a-3p expression by binding to m^6^A-modified RNA, thereby inhibiting SMAD5 activity and delaying osteogenic differentiation and fracture healing [168]. IGFBP3 also inhibits osteogenic differentiation by reducing the expression of key osteogenic markers and attenuating BMP-2-induced ALP activity [169]. Furthermore, IGFBP3 overexpression inhibits osteogenic differentiation and significantly reduces the proliferative capacity of ADSCs [170]. Similarly, miR-23a-3p directly targets SMAD5, downregulates its protein expression, and inhibits osteogenic differentiation [171].

YTHDF1 expression is significantly upregulated during osteogenic differentiation of BMSCs. Increased YTHDF1 enhances cellular proliferation and osteogenic differentiation, whereas reduced YTHDF1 exerts opposing effects. Autophagy activity is also a critical contributing factor. *In vivo* experiments have validated that elevated YTHDF1 expression accelerates fracture healing and improves bone microarchitecture [133]. Conversely, YTHDF1 deficiency diminishes the osteogenic differentiation capacity of human BMSCs (hBMSCs) *in vitro*. The protein zNF839/Zfp839 functions as a downstream target of YTHDF1 and promotes BMSC osteogenic differentiation through m^6^A-dependent translational regulation and interaction with Runx2 [134].

Besides, activation of the Wnt/β-catenin signaling pathway during chondrogenic differentiation markedly increases YTHDF1 expression, thereby promoting cartilage formation in hBMSCs. Altered YTHDF1 expression, either through overexpression or knockdown, substantially affects chondrogenesis by enhancing cartilage matrix synthesis while inhibiting the expression of cartilage markers [172]. Under hypoxic conditions, YTHDF1 enhances thrombospondin 1 (*THBS1*) mRNA stability to promote osteogenic differentiation. Silencing of YTHDF1 or THBS1 exacerbates hypoxia-induced inhibition of osteogenic differentiation. These findings indicate that the YTHDF1/THBS1 signaling pathway may mitigate the inhibitory effects of hypoxia on osteogenic differentiation [135].

#### Evidence appraisal, knowledge gaps, and translational relevance-bone

Evidence supporting the role of METTL3 and YTHDF1 in osteogenesis is robust across diverse *in vitro* systems and *in vivo* fracture models [135, 149, 172]. However, evidence derived from human callus tissue remains limited.

Convergence: YTHDF1 promotes osteogenic stability under hypoxic conditions through THBS1-mediated mechanisms that counteract differentiation impairment [135].

Contradictions: METTL3 plays a dual, context-dependent role in osteogenesis, promoting or inhibiting osteogenic differentiation according to the inflammatory signaling conditions and target specificity [131, 149].

Gaps: (i) Spatial single-cell mapping is required to define RNA modification dynamics during the transition from hematoma to callus and subsequent remodeling; (ii) development of bone-targeted delivery systems for m^6^A editors and readers is necessary; (iii) validation studies using large animal models are required to enable comparative assessment.

Limitations and Species Differences: Murine skeletal physiology and gene regulatory networks differ from those of humans. Consequently, most mechanistic studies rely on gene knockdowns and overexpression approaches. The differential functional outcomes of key regulators, such as the pronounced osteogenic phenotype induced by METTL3 manipulation compared with the more limited effect of targeting FTO or ALKBH5 in specific contexts [147, 157], underscore the need for human-based studies and rigorous validation of therapeutic targets.

Translational Relevance: Delivery of mRNA or modulators of RNA-modification, as well as bone-targeted interventions addressing hypoxia or inflammation, may enhance callus formation. Imaging and biomechanical assessments will be critical for evaluating outcomes.

Collectively, these findings indicate that m^6^A modifications regulate molecular events underlying osteogenic differentiation, bone metabolism, and the bone microenvironment through a dynamic network of writers, erasers, and reader proteins, thereby influencing fracture healing. Post-transcriptional regulation of m^6^A methylation signaling plays a central role in bone regeneration and provides a molecular foundation for m^6^A-targeted therapeutic strategies with high translation potential.

### The role of *N^6^*-methyladenosine modifications in corneal wound healing

#### Corneal anatomy and the imperative for wound repair

The cornea, a transparent and avascular tissue, constitutes the primary structural barrier of the eye. It consists of cellular and non-cellular components, including epithelial, stromal, and endothelial cells. The cornea’s anatomical position and barrier function render it highly susceptible to mechanical injury and pathogen invasion [173]. Effective healing of corneal lesions is essential to preserve visual function and maintain tissue integrity [174]. Corneal wound healing is a complex biological process involving cell death, migration, proliferation, differentiation, and ECM remodeling. Epithelial and endothelial injury are the most common initiators of this process, although immune-mediated or infectious injuries accessing the stroma via corneal limbal vessels may also contribute [74, 175].

The corneal epithelium possesses continuous self-renewal under physiological conditions, maintaining tissue integrity and transparency. Following injury, wound-healing responses are essential to prevent microbial invasion of the stroma, which may result in secondary injury [176]. Untreated corneal injuries can substantially impair visual function and may lead to inflammation, neovascularization, ulceration, and scarring [177].

#### *N^6^*-methyladenosine machinery in corneal epithelial repair, fibrosis, and neovascularization

Recent research has demonstrated that m^6^A RNA methylation plays a critical role in corneal wound healing. m^6^A-related components, including METTL3, FTO, and YTHDF3, are extensively involved in regulating corneal biological processes such as cell proliferation, migration, fibrotic response, and neovascularization, underscoring their essential role in corneal injury repair [178]. *In vitro* studies demonstrate that METTL3 silencing or overexpression markedly influences endothelial cell viability, proliferation, migration, and tube formation [179]. Furthermore, corneal fibrotic responses are substantially modulated by m^6^A modification and associated regulators.

**Figure 5:**
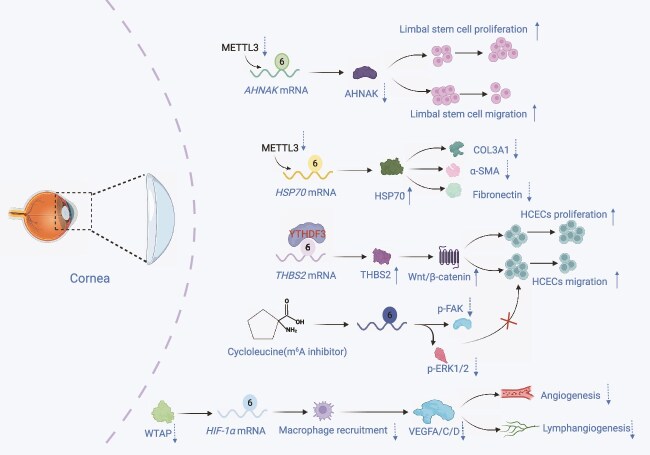
Role of m^6^A modification in corneal wound healing. Corneal injury induces dynamic changes in m^6^A modification that coordinate epithelial regeneration and stromal remodeling. METTL3 exerts context-dependent effects: in corneal limbal stem cells, METTL3 suppression enhances proliferation and migration by reducing m^6^A modification of *AHNAK* and *DDIT4* transcripts, whereas in fibrotic responses, METTL3 upregulation correlates with increased α-SMA and fibronectin expression. FTO silencing has minimal impact on fibrosis markers, indicating pathway-specific roles of writers versus erasers in corneal pathology. WTAP modulates CNV by regulating *HIF-1α* translation efficiency via m^6^A, thereby controlling VEGFA/C/D secretion from infiltrating macrophages. In diabetic CEWH, YTHDF3 binds to m^6^A sites on *THBS2* mRNA, enhancing translation and activating Wnt/β-catenin signaling to promote epithelial regeneration. The m^6^A inhibitor cycloleucine delays CEWH by suppressing p-FAK and p-ERK1/2 signaling. *CNV* corneal neovascularization, *CEWH* corneal epithelial wound healing, *METTL3* methyltransferase-like 3, *HSP70* heat shock protein 70, *YTHDF3* YTH *N*^6^-methyladenosine RNA binding protein F3, *THBS2* thrombospondin 2, *HIF-1α* hypoxia-inducible factor 1-alpha, *VEGF* vascular endothelial growth factor, *HCECs* human corneal endothelial cells. Figure created with BioRender.com (licenses: DR296U1HUR).

Upregulation of m^6^A levels and the expression of regulators METTL3 and FTO in corneal fibrosis models induced by alkali burn or transforming growth factor-beta 1 (TGF-β1) correlates with increased expression of fibrotic markers. Silencing of the writer METTL3 reduced fibrosis, evidenced by decreased levels of COL3A1, α-SMA, and fibronectin, along with an increase in the protective heat shock protein 70 (HSP70). In contrast, silencing of the eraser FTO exerted no effect in this context [139] (Figure 5). These findings indicate that the m^6^A machinery exhibits high specificity and potential functional redundancy. Most data derive from short-term studies focused on epithelial endpoints, and their implications for stromal fibrosis and long-term corneal transparency require further *in vivo* investigation.

Investigation of m^6^A modification during corneal alkali burn repair revealed that the m^6^A methyltransferase METTL3 plays a critical role in corneal injury repair via corneal limbal stem cells. METTL3 regulates the expression levels of AHNAK and DDIT4. METTL3 suppression in corneal limbal stem cells significantly enhanced cell proliferation and migration, facilitating corneal repair. RNA sequencing and m^6^A profiling demonstrated that m^6^A modification levels of AHNAK and DDIT4 significantly decreased upon METTL3 knockdown, indicating that AHNAK and DDIT4 may be m^6^A target genes of METTL3 [73]. Furthermore, WTAP is involved in angiogenesis and lymphangiogenesis during corneal neovascularization (CNV) by modulating macrophage recruitment and VEGF secretion via m^6^A modification. WTAP enhances the translational efficiency of HIF-1α, which in turn induces macrophage secretion of VEGF-A, -C, and -D. *In vivo* studies revealed that WTAP suppression restricted macrophage recruitment and VEGF secretion, thereby hindering angiogenesis and lymphangiogenesis in CNV [137].

In addition, m^6^A RNA modification increases markedly during corneal epithelial wound healing (CEWH) in mice, where it regulates corneal epithelial cell proliferation and migration, thereby promoting healing. The m^6^A inhibitor cycloleucine significantly delayed CEWH by inhibiting the proliferation and migration of human corneal epithelial cells (HCECs) and reducing the protein levels of p-FAK and p-ERK1/2 [180]. The m^6^A reader protein YTHDF3 facilitates translation of THBS2 by binding to the m^6^A site in *THBS2* mRNA, activating the Wnt/β-catenin signaling pathway and accelerating diabetic CEWH (Figure 5). In diabetic mice, THBS2 expression is downregulated; its overexpression markedly enhances HCEC proliferation and migration, supporting corneal epithelial regeneration [138].

#### Evidence appraisal, knowledge gaps, and translational relevance in cornea

Strength of Evidence: *In vivo* studies using corneal injury and diabetic models demonstrate the critical roles of METTL3, WTAP, and YTHDF3 [73, 137, 180]. Nonetheless, studies in humans are largely limited to *ex vivo* analyses or small cohort investigations.

Convergence: YTHDF3 enhances epithelial healing in the diabetic cornea via THBS2 [138].

Divergence: METTL3 knockdown reduces fibrosis markers, whereas FTO knockdown exerts minimal effects in certain models [139], indicating selective pathway dependencies.

Gaps: (i) Phase-specific studies are required to differentiate epithelial and stromal responses, separating re-epithelialization from fibrosis; (ii) investigations are needed to define optimal topical delivery windows and associated safety biomarkers.

Limitations and Species Differences: Mouse models may not fully replicate human immune privilege and tissue reconstruction. Most studies focus on early epithelial endpoints, whereas stromal fibrosis and transparency require longer-term follow-up. The minimal impact of FTO knockdown—potentially due to reader/writer redundancy in certain fibrosis paradigms, such as METTL3 silencing—highlights the need for site-specific validation and temporal control.

Translational Relevance: Modulation of the YTHDF3–THBS2 axis on the ocular surface may accelerate diabetic corneal closure while minimizing interstitial fibrotic scarring. However, precise calibration of expression levels will be essential.

Therapeutic strategies targeting m^6^A RNA methylation and key regulators, including METTL3, WTAP, and YTHDF3, represent a promising approach. Such interventions may enhance corneal wound healing by regulating fundamental pathological processes, such as epithelial repair, angiogenesis, and fibrosis.

#### Common and tissue-specific mechanisms of *N^6^*-methyladenosine modification across wound types

Despite distinct etiologies and healing trajectories, diabetic ulcers, burn injuries, fractures, and corneal wounds share m^6^A as a central post-transcriptional regulator. Elucidating the mechanism by which m^6^A orchestrates conserved and tissue-specific repair programs could inform the development of targeted therapeutics.

#### Conserved mechanisms

**Figure 6:**
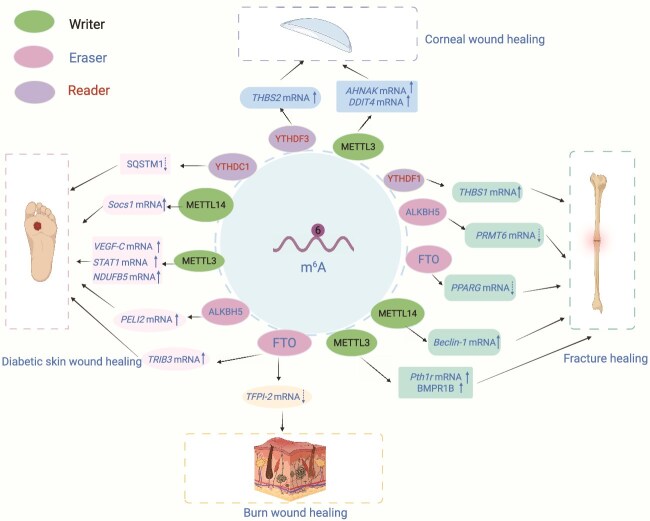
Multifaceted regulatory network of m^6^A RNA methylation in tissue repair. This schematic illustration summarizes the m^6^A-dependent epitranscriptomic landscape across four wound healing contexts: diabetic skin wounds, burn wounds, fractures, and corneal injuries. The m^6^A MTC (writers: METTL3 and METTL14) catalyzes adenosine methylation on target transcripts, whereas demethylases (erasers: FTO and ALKBH5) dynamically remove these marks, enabling reversible, context-specific gene regulation. Reader proteins (YTHDF1, YTHDF3, and YTHDC1) recognize m^6^A-modified RNAs and mediate downstream effects on mRNA stability, translation, and nuclear export. In diabetic skin wound healing, METTL3 promotes m^6^A methylation of *VEGF-C*, *STAT1*, and *PELI2* mRNAs, modulating lymphangiogenesis, macrophage polarization, and keratinocyte migration, respectively; YTHDF1 recognizes methylated *NDUFB5* mRNA to regulate mitochondrial function; and ALKBH5 demethylates SQSTM1 transcripts, influencing autophagy and cell survival. In burn wound healing, FTO demethylates *TFPI-2* mRNA to accelerate re-epithelialization, while METTL3 regulates PPARG and TRIB3 expression to balance inflammation and tissue remodeling. In fracture healing, METTL14 methylates *Pth1r* and *BMPR1B* mRNAs to enhance osteogenic differentiation; FTO demethylates PRMT6 transcripts to fine-tune bone regeneration; and YTHDC1 modulates *Beclin-1* mRNA nuclear export, linking autophagy to repair. In corneal wound healing, METTL3 destabilizes *AHNAK* and *DDIT4* mRNA to expand limbal stem cells; YTHDF3 recognizes *THBS2* and *THBS1* mRNAs, activating Wnt/β-catenin and Socs1-mediated pathways for epithelial regeneration. *METTL3* methyltransferase-like 3, *METTL14* methyltransferase-like 14, *FTO* fat mass and obesity-associated protein, *ALKBH5* ALKB homolog 5, *YTHDF1* YTH *N^6^*-methyladenosine RNA binding protein F1, *YTHDF3* YTH *N^6^*-methyladenosine RNA binding protein F3, *YTHDC1* YTH domain-containing 1, *THBS1* thrombospondin 1, *VEGF-C* vascular endothelial growth factor C, *STAT1* signal transducer and activator of transcription 1, *NDUFB5* NADH:ubiquinone oxidoreductase subunit B5, *Socs1* suppressor of cytokine signaling 1, *TFPI-2* tissue factor pathway inhibitor 2, *SQSTM1* sequestosome 1, *Pth1r* parathyroid hormone 1 receptor, *THBS2* thrombospondin 2. Figure created with BioRender.com (licenses: CS296U1RGE).

The core m^6^A machinery—writers, erasers, and readers—exhibits functional conservation across various tissues, coordinating proliferation, migration, and differentiation during repair. METTL3 serves as a regulatory hub with tissue-specific effects. In diabetic skin wounds, METTL3 promotes lymphangiogenesis by stabilizing *VEGF-C* mRNA via IGF2BP2 [108]. During fracture repair, it drives osteogenic differentiation by methylating Pth1r or modulating BMPR1B through competing endogenous RNA mechanisms [131, 132]. In corneal epithelium, METTL3 targets AHNAK and DDIT4 to regulate migration and proliferation (Figure 6) [73].

Beyond cell-fate specification, m^6^A regulates inflammation across tissues. In diabetic wounds, METTL3 promotes M1 macrophage polarization via STAT1 [97], whereas METTL14 drives M2 polarization through the YTHDF1–Socs1 axis [94]. Comparable macrophage programming occurs during fracture healing [158]. The (lymph)angiogenic machinery also exhibits cross-tissue conservation: METTL3 and WTAP consistently modulate VEGF family members in skin, bone, and cornea [108, 137, 156], establishing m^6^A as a central regulator of vascular remodeling.

#### Tissue-specific programs

Cellular composition, microenvironmental cues, and functional demands confer tissue specificity to m^6^A regulation. Individual reader proteins exemplify this context-dependence. YTHDC1 modulates autophagy in diabetic keratinocytes by targeting the receptor SQSTM1 [104], whereas YTHDF1 enhances translation of osteogenic factors (ZNF839 and THBS1) in BMSCs during fracture repair [133–135]. This divergence reflects tissue priorities: metabolic adaptation in skin versus lineage commitment in bone (Figure 6).

Tissue-specific gene networks further define m^6^A outcomes. Bone healing relies on the BMP/SMAD and the Wnt/β-catenin pathways, in which m^6^A modulation of master transcription factors RUNX2 and PPARG facilitates a balance between osteogenic and adipogenic fates [136, 147]. Corneal repair emphasizes transparency and antifibrotic programs, with m^6^A suppressing COL3A1 and α-SMA [139], and inhibiting pathological neovascularization through HIF-1α/VEGF regulation, a critical mechanism in this avascular tissue [137]. Skin repair prioritizes barrier restoration, with m^6^A promoting epithelial migration via PELI2 and SPARC [16, 105], thereby contributing to re-epithelialization and appendage regeneration.

#### Implications

m^6^A methylation functions as a regulatory mechanism that calibrates universal repair programs with tissue-specific responses. Key regulators, such as METTL3, coordinate these processes at a global level, while local microenvironmental cues constrain target selection and determine functional outcomes. The presence of a conserved m^6^A machinery with diverse tissue-dependent outputs underscores the need for therapeutic strategies that account for this complexity. Accordingly, effective interventions must integrate systemic and tissue-specific considerations to optimize outcomes. Future RNA-based strategies are expected to leverage this knowledge to achieve greater precision, either by targeting shared regulatory nodes to benefit multiple tissues, such as the heart and kidney, or by exploiting tissue-specific dependencies to enable targeted repair with minimal off-target effects.

### Effects of other ribonucleic acid modifications in wound healing

Although m^6^A is a major regulatory modification, epitranscriptomic control of wound healing involves a broader and more complex network of RNA modifications. Additional marks, including ac^4^C, m^7^G, and m^5^C, constitute important layers of post-transcriptional regulation beyond m^6^A.

#### *N^4^*-acetylcytidine in cellular migration and differentiation

Emerging evidence indicates that NAT10 mediates cytosine-4 acetylation and contributes to tissue repair. NAT10 functions as a key regulator of keratinocyte migration during skin wound healing by stabilizing mRNAs encoding IL-6, IL-8, and NF-κB/p65, thereby accelerating healing. Conversely, NAT10 deficiency disrupts this regulatory cascade, reduces NF-κB/p65 activity and cytokine expression, and promotes nuclear degradation of p65, collectively delaying re-epithelialization [20].

NAT10-mediated ac^4^C modification broadly promotes vascular and skeletal repair by enhancing mRNA stability and cellular differentiation. In injured arteries, NAT10 activates FAK signaling to drive vascular smooth muscle cell proliferation and vascular remodeling, whereas in MSCs, it supports osteogenic differentiation through ac^4^C-dependent regulation of *Gremlin 1* and *RUNX2* mRNAs. Notably, inflammatory suppression of NAT10 activity impairs BMSC osteogenesis, an effect that is reversed by restoring acetyl-coenzyme A availability through sodium citrate supplementation or ATP citrate lyase activation [77–80]. In addition, mouse amniotic fluid-derived MSCs stimulate corneal epithelial cell proliferation and exert anti-inflammatory effects via the ETV4/JUN/CCND2 axis and increased ac^4^C modification of mRNA [181].

#### *N^7^*-methylguanosine and its role in angiogenesis and bone regeneration

m^7^G modification, catalyzed by the primary methyltransferase METTL1, is emerging as a key regulator of vascularization and tissue regeneration. Although conventional unmodified mRNA possesses inherent therapeutic potential, mRNA-modified exhibits enhanced efficacy. Similarly, functional studies in bone defect models have demonstrated that bone-targeted lipid nanoparticles (LNPs) carrying m^7^G-modified *Runx2* mRNA substantially enhance bone repair and regeneration, offering a promising strategy for regenerative medicine [75]. METTL1 also promotes blood flow recovery and angiogenesis by upregulating *VEGFA* mRNA translation in ischemic tissues. *In vitro* analyses indicated that increased METTL1 expression considerably promotes proliferation, migration, and angiogenic potential in HUVECs [76].

#### 5-methylcytosine in stem cell function and tissue homeostasis

The m^5^C modification is a key component of the epitranscriptomic network and contributes to the regulation of wound healing. RNA methyltransferase NSUN2, a principal m^5^C writer, is essential for somatic stem cell homeostasis and plasticity. Hair follicle stem cells consistently express NSUN2, which is required for their timely activation and proper differentiation. Absence of NSUN2 expression prolongs quiescence and induces aberrant lineage commitment, thereby disrupting the hair follicle regenerative cycle [182].

Furthermore, NSUN2 promotes CEWH via m^5^C modification. NSUN2 knockdown inhibits CEWH and the proliferation and migration of HCECs, whereas its overexpression produces the opposite effects. During CEWH, NSUN2 expression and RNA m^5^C modification levels markedly increase. NSUN2 interacts with the Aly/REF export factor through m^5^C modification to enhance UHRF1 translation. Studies demonstrate that UHRF1 inhibition blocks CEWH and HCEC function, whereas UHRF1 overexpression rescues the effects of NSUN2 silencing [21]. Moreover, YBX1 promotes angiogenesis-dependent osteogenesis through m^5^C-dependent mechanisms, and its deletion destabilizes markers such as CD31, EMCN, and BMP4, thereby inhibiting osteogenic differentiation of bone mesenchymal stromal cells [183].

#### Epitranscriptomic crosstalk and the future of ribonucleic acid therapeutics

Accumulating evidence indicates that RNA modifications do not operate in isolation but undergo functional crosstalk that coordinates tissue remodeling. In pathological skin fibrosis, ALKBH3-mediated m^1^A demethylation elevates METTL3 expression by preventing YTHDF2-dependent degradation of *METTL3* mRNA; increased METTL3 activity subsequently reinforces m^6^A-dependent stabilization of ECM transcripts, including COL1A1 and FN1, ultimately driving fibrotic progression [184]. This mechanism illustrates a unique crosstalk between m1A and m^6^A, indicating that RNA modifications are not independent and are governed by a complex, coordinated regulatory network.

Studies on ac^4^C, m^7^G, m^5^C, pseudouridine, and m^1^A demonstrate a multilayered epitranscriptomic network that regulates wound healing across the inflammation, proliferation, and remodeling phases. NAT10-dependent ac^4^C enhances the stability of pro-repair transcripts in skin, vasculature, bone, and cornea [77–80]. Therapeutic mRNAs incorporating METTL1-mediated m^7^G promote angiogenesis and osteogenesis [75, 76]. NSUN2-regulated m^5^C modifications modulate epithelial and stem cell changes in skin and cornea and connect vascular signals to bone development [21, 182, 183]. Furthermore, DKC1-mediated m^1^ψ pathways stimulate cytokine and VEGF-A expression and promote tissue regeneration [185, 186]. These findings suggest that crosstalk between modifications, including m^1^A and m^6^A, can determine regenerative versus fibrotic outcomes [184]. Future research integrating site-resolved mapping with mechanistic validation of writer–reader–eraser axes, along with phase-specific delivery of modified RNAs, is essential to accelerate the translation of these findings into precision therapies for complex wounds.

### Translational outlook for burns and trauma

#### Candidate therapeutic strategies and delivery routes

##### Skin (diabetic foot ulcers, burn)

The most advanced approaches for skin wound healing involve localized epitranscriptomic manipulation to target the key pathophysiological processes underlying impaired healing [187]. Hydrogels incorporating FTO inhibitors that scavenge ROS represent a promising strategy, as they concomitantly target oxidative stress, chronic inflammation, and defective re-epithelialization observed in DFU and burn wounds. Antioxidant hydrogel matrices composed of catechol-modified polysaccharides, hyaluronic acid, and polydopamine scaffolds deliver nanoscale FTO inhibitor formulations at 0.1%–1.0% w/w and provide adhesive, breathable properties with optimal moisture retention [188]. When applied topically to DFU margins and wound base daily or every 48 h as adjunctive therapy, these hydrogels accelerate re-epithelialization to 100%, shorten healing time, reduce infection rates, and decrease pain scores. Nucleoside modifications in wound exudate cells, assessed by liquid chromatography–tandem mass spectrometry (LC–MS/MS) and MeRIP–qPCR/miCLIP2 profiling of sentinel transcripts from debrided edge biopsies at baseline, week 2, and closure, confirm target engagement [32]. Close monitoring for delayed granulation or premature excessive angiogenesis is critical for safety, with offloading and debridement remaining standard care in DFUs.

Exosomes from METTL3-modified ADSCs have been developed to exploit the inherent regenerative properties of MSC-derived extracellular vesicles (EVs) while delivering cargo that enhances m^6^A-mediated repair programs [189]. Manufacturing protocols involve culturing ADSCs in serum-free medium, optionally followed by transient METTL3 modulation. Purification is achieved by ultrafiltration combined with size-exclusion chromatography or density-gradient ultracentrifugation, yielding preparations devoid of protein aggregates and lipoproteins [190]. Release criteria include assessment of particle size and concentration by nanoparticle tracking analysis, identification via CD63/CD81/CD9 expression, quantitation of cargo (*METTL3* mRNA and protein levels), and evaluation of functional activity using fibroblast migration and HUVEC tube formation assays. Standard dosing consists of 1–5 × 10^9^ particles per cm^2^ of wound area, administered by spray or gel matrix once daily or every 48 h for 1–2 weeks, with monitoring for local inflammation, fibrosis, and scarring [191].

NAT10-mediated ac^4^C modulation constitutes a third therapeutic strategy, particularly relevant during the critical transition from inflammation to proliferation [65]. NAT10-driven ac^4^C enhances mRNA stability and translation and intersects with NF-κB/IL-6/IL-8 signaling programs that regulate keratinocyte migration and early wound repair. This approach requires strict temporal control, with a brief treatment window limited to days 2–6 post-injury and low topical dosing; treatment should be discontinued immediately upon the appearance of hypertrophic scarring [192]. Pharmacodynamic evaluation includes ac^4^C quantification by LC–MS/MS, analysis of NAT10 target mRNA stabilization in wound-edge biopsies, and concurrent monitoring of scar indices, pruritus, and early collagen III/I ratios.

##### Bone fracture therapeutics

Bone-targeted LNPs delivering osteogenic mRNA payloads, including *RUNX2, SP7/OSX, or BMP2* surrogates, address the therapeutic needs of osteoporotic and delayed-union fractures through transient, controllable osteogenic stimulation [193]. These formulations employ bisphosphonate- or acidic peptide-decorated lipids to localize payloads at mineralized surfaces, with the LNP composition adhering to the gene therapy CMC guidance for ionizable lipid, helper lipid, cholesterol, and PEG-lipid ratios [194, 195]. Administration typically consists of local perifracture injection at the time of fixation or during the early callus phase (days 3–10) to synchronize with proliferative osteogenesis. Primary endpoints include time to radiographic union and quantitative callus architecture assessed by low-dose computed tomography (CT) for trabecular thickness and mineral density, while secondary outcomes encompass patient-reported pain and function scores, weight-bearing milestones, and biochemical markers such as serum ALP and osteocalcin. Safety monitoring requires vigilance for off-target mineralization using imaging and laboratory assessments [196].

Combination strategies targeting hypoxia-associated pathways, particularly YTHDF1-dependent stress translation programs, can be integrated with oxygenation interventions, including normobaric or hyperbaric oxygen therapy, and anti-inflammatory regimens. This multimodal strategy mitigates impaired osteogenesis characteristic of highly inflammatory and hypoxic microenvironments and may accelerate fracture healing in complex cases involving compromised vascular supply or systemic metabolic dysfunction [197].

##### Cornea

YTHDF-enhanced epithelial repair programs promote resource prioritization in diabetic corneal wounds in a manner analogous to preconditioning, with the THBS2 axis serving as a critical regulatory input. According to the treatment protocol, topical application is performed every 6 to 12 h for 3 to 5 days following injury, with unilateral dosing and the contralateral eye serving as control when ethically permissible. Efficacy assessment is based on fluorescein staining patterns, measurement of epithelial thickness by optical coherence tomography (OCT) [198], and graded corneal haze on a scale of 0–4. Safety evaluation includes monitoring for photophobia, progression of corneal haze, neovascularization, alterations in intraocular pressure, and infections [199].

Modulation of macrophage VEGF programs through WTAP and HIF-1α may selectively promote epithelial recovery while limiting pathological stromal neovascularization in the diabetic cornea [200]. This approach employs short-course topical or subconjunctival administration, supported by cautious ascending dose-finding studies and predefined anti-VEGF rescue criteria in the event of early neovascularization signals [201]. Endpoints emphasize quantification of corneal neovascular area using slit-lamp photography with image analysis, assessment of haze progression, and evaluation of best-corrected visual acuity when applicable [202, 203].

#### Diagnostics and pharmacodynamic biomarkers

Circulating and exosomal epitranscriptomic signatures serve as systemic biomarkers for stratifying wound chronicity and monitoring therapeutic response. Plasma or serum extracellular vesicle RNA is analyzed for global m^6^A levels and site-specific modifications on key wound transcripts using LC–MS/MS and targeted MeRIP–qPCR or miCLIP2 assays [204]. Sampling follows a structured cadence, including baseline assessment, week 1–2 monitoring, and closure or standardized time points for burns and corneal wounds. Analytical protocols employ synthetic spike-ins and replicate libraries, with normalization to EV counts by nanoparticle tracking analysis and total RNA content. Primary pharmacodynamic transcripts are predefined across inflammation, angiogenesis, and ECM remodeling pathways, supporting time-staged dosing optimization and predictive response assessment [205].

Using these matrices, LC–MS/MS quantification of modified nucleosides (proxies for NAT10 and METTL3 activity) and, when technically feasible, reader occupancy can be conducted. Validation of these correlations establishes links between pharmacodynamic alterations and closure rate as well as histological parameters in patient cohorts undergoing debridement or therapeutic keratectomy. Standardization considerations include the timing of collection, the sample acquisition method, optimal storage temperature, and limitations on freeze–thaw cycles [206].

#### Trial design considerations for burns and trauma

Clinical trials for adjunctive therapy in DFUs employ randomized, controlled designs, with treatment administered in addition to standard care, including offloading, debridement, and infection management. Block randomization stratified by ulcer size and Wagner grade ensures balanced allocation across treatment groups. Outcome assessors remain blinded to group allocation of patients. Patient enrichment criteria include HbA1c levels of 7%–10%, ulcer sizes between 1 and 10 cm^2^, ulcer duration exceeding 4 weeks, adequate perfusion (ankle-brachial index ≥0.7), and absence of active osteomyelitis. The primary endpoint, time to complete (100%) re-epithelization, is complemented by key secondary measures, including the incidence and timing of first infection, amputation-free survival, pain score, scar quality assessed by POSAS or Vancouver scales, and ulcer recurrence at 12–24 weeks. Sample sizes are calculated to provide 80%–90% power to detect a 20%–25% reduction in median time-to-closure with two-sided α = 0.05, with analyses stratified by neuropathic and ischemic DFU subtypes. Futility and safety interim analyses are conducted under the oversight of the Data Safety Monitoring Board [207, 208].

Burn partial-thickness wound trials are typically performed using split-site or intra-patient randomized designs to evaluate topical or EV therapies. In the trials, the debridement and dressings are standardized. Blinded planimetry and scar scale assessments are conducted [209]. Endpoints include time to re-epithelialization, Vancouver Scar Scale or POSAS scores at 3 and 6 months, infection incidence, and patient-reported pain and pruritus scores. Serial high-resolution photography under controlled light conditions, with a color calibration chart optionally included to enable objective color assessment. Imaging may be complemented by laser Doppler or thermal techniques to evaluate perfusion [210].

The primary endpoint is time to epithelial closure, determined by fluorescein staining. Secondary endpoints include OCT-derived epithelial thickness recovery, haze grading, best-corrected visual acuity, and intraocular pressure. According to the safety stopping criteria, threshold changes in haze, early neovascularization, or persistent epithelial defects beyond predefined limits mandate trial interruption [211].

Statistical methods specify estimands for intercurrent events such as rescue antibiotics or early grafting, employ mixed-effects models for repeated measures, and utilize time-to-event analyses incorporating competing risks [212].

#### Safety and regulatory considerations

Local delivery strategies via topical skin or ocular application and locally injected bone-targeted LNPs minimize systemic exposure and off-target organ risks while preserving therapeutic efficacy. Biodistribution and shedding studies are essential for EVs and LNPs, with specific evaluation of complement activation-related pseudoallergy for nanoparticulate formulations. Anticipated off-target effects require systematic monitoring: fibrosis and aberrant lymphangiogenesis through scar scales, ultrasound elastography for skin, and slit-lamp angiography for cornea; ectopic or heterotopic mineralization via ALP activity, calcium and phosphate panels, and low-dose CT for off-target calcifications; and immunogenicity through anti-PEG antibody titers for LNPs and cytokine panels combined with ocular inflammation grading. Although genotoxicity is not expected for non-integrating mRNA or EV approaches, standard safety pharmacology and local tolerance studies following ICH S9 and S10 guidelines remain appropriate for regulatory submissions [213].

Chemistry, manufacturing, and control readiness require phase-appropriate characterization. EV products require batch-to-batch consistency in size and particle number, identity verification via tetraspanin markers, purity assessment for protein and lipoprotein contaminants, potency assays aligned with mechanisms of action, such as migration or angiogenesis, sterility and endotoxin testing, and residual DNA/RNA quantification [214]. LNP formulations must have defined compositions and ratios, confirmed encapsulation efficiency, mRNA capping, and poly(A) tailing, stability under accelerated and real-time conditions, release potency demonstrated through *in vitro* and *in vivo* protein expression, and degradant profiling [215]. All characterization and control measures should comply with ISEV/MISEV standards for EV analytics and FDA/EMA guidance for LNP gene therapy CMC packages, including process controls, comparability, and release testing [216].

Following preclinical validation of epitranscriptomic therapies for burns and trauma, this review proposes their clinical translation with rigorous safety monitoring throughout all stages of clinical development. This strategy emphasizes targeted delivery systems, phase-appropriate biomarkers, and trial designs tailored to minimize toxicity and address distinct wound types.

### Challenges and future perspectives

This review systematically synthesizes current advances in RNA modifications across multiple wound healing contexts. RNA modification has emerged as a promising therapeutic target and constitutes a central regulatory mechanism in tissue growth, development, and homeostasis. Analysis of the existing literature indicates that research to date has primarily focused on cutaneous injuries and fracture healing, with comparatively limited investigation of corneal and other trauma-related wounds.

Despite notable advances in defining the therapeutic potential of RNA modifications in wound healing, the field remains limited by critical knowledge gaps, methodological constraints, and a substantial translational divide. Progressing this field from mechanistic insights to clinical application will require coordinated and multifaceted strategies.

#### Knowledge gaps and context-dependency

The majority of current research focuses on m^6^A, whereas other RNA modifications, including m^5^C, m^7^G, and ac^4^C, remain largely unexplored within specific wound contexts. Recent studies have begun to associate these modifications with repair processes, such as NSUN2-mediated m^5^C modification of UHRF1 in corneal epithelial migration [21], METTL1-mediated m^7^G modification of VEGFA during angiogenesis [76], and NAT10-mediated ac^4^C modification of ITGB1 during vascular repair [77]. However, their precise roles in diabetic ulcers, burns, and fractures remain poorly defined.

Even within m^6^A-focused studies, strong context dependency has produced contradictory findings that hinder translational advances. For instance, in diabetic wounds, the demethylase FTO has been reported to either impair or accelerate healing by demethylating TRIB3 [100]. This apparent contradiction underscores that the function of epitranscriptomic regulators is determined by cell type, disease stage, and microenvironment.

#### The challenge of cellular and spatiotemporal heterogeneity

Wound beds constitute complex and dynamic ecosystems. Although recent studies have started to examine the roles of m^6^A in keratinocytes and fibroblasts [108], its regulatory functions in other critical cellular players remain poorly understood. m^6^A programming exerts cell-type-specific effects on macrophage polarization [97], adaptive immune responses, and endothelial cell behavior. The temporal coordination among writers, erasers, and readers, and their crosstalk with ncRNAs, including circ-Amotl1 [106], can be conceptualized as a regulatory network whose systems-level significance remains undefined.

#### Bridging the bench-to-bedside chasm

The principal obstacle remains a substantial translation gap. Most existing evidence derives from immortalized cell lines and rodent models [90, 91], which fail to accurately replicate human tissue repair physiology due to fundamental differences in skin architecture, immune activity, and healing kinetics [140, 141]. Consequently, promising strategies such as nano-hydrogel-based FTO inhibitors and METTL3 modulators [96, 99] remain confined to preclinical validation. Human safety profiles, pharmacodynamic properties, and long-term efficacy have yet to be established.

#### A path forward: technological innovation and translational priorities

A major limitation of current epitranscriptomic research in wound healing is reliance on bulk tissue analyses, which obscure the cellular heterogeneity intrinsic to the wound microenvironment. To overcome this limitation, the field must adopt emerging single-cell and spatial epitranscriptomic technologies that enable analysis of RNA modification landscapes at unprecedented cellular resolution.

Several methodologies merit focused attention. Single-cell m6A sequencing and DART-seq enable transcriptome-wide m^6^A profiling at the individual-cell level, facilitating the characterization of modification heterogeneity among keratinocytes, fibroblasts, immune cells, and endothelial populations within healing wounds [217–219]. Complementary strategies, including single-cell SLAM-seq combined with m^6^A immunoprecipitation, permit concurrent evaluation of RNA synthesis dynamics and modification status, thereby elucidating temporal patterns of epitranscriptomic regulation across distinct phases of wound healing. Integration of single-cell RNA sequencing with computational prediction tools, such as m^6^A-Atlas and SRAMP, provides additional capacity to infer modification landscapes when direct detection remains technically challenging. Furthermore, spatial transcriptomics platforms, including Visium, MERFISH, and Xenium, are increasingly being adapted to incorporate epitranscriptomic readouts, enabling *in situ* mapping of RNA modifications within the structural context of wound tissue. This capability is essential for understanding molecular regulation at the wound edge, within granulation tissue, and across re-epithelialization fronts [220–223].

Beyond technological innovation, the field must address fundamental conceptual and methodological gaps. Application of these approaches to wound healing models can address several critical questions: (1) How do m^6^A landscapes differ between pro-regenerative and pro-fibrotic fibroblast subpopulations? (2) What epitranscriptomic signatures distinguish M1 and M2 macrophage polarization states in diabetic and non-diabetic wounds? (3) How do RNA modification dynamics evolve spatially from the wound margin to the healing center? In parallel, systematic investigation of modifications beyond m^6^A—including m^5^C, m^7^G, ac^4^C, and pseudouridine—is required to construct comprehensive dynamic atlases across human wound types and healing phases and to identify novel therapeutic targets.

To enhance preclinical model fidelity, methodological approaches must adopt human primary cells, patient-derived organoids, and tissue-specific conditional knockout animals that more accurately replicate the complex microenvironment of human wounds. These models are especially critical for diabetic and burn wound research, where species-specific differences in skin architecture and inflammatory responses constrain the translational applicability of conventional rodent studies.

For clinical translation, developing tissue- and cell-specific delivery platforms—particularly nanocarrier-based systems—is essential to optimize therapeutic efficacy while minimizing off-target effects. All candidate interventions should undergo rigorous preclinical evaluation and subsequent carefully designed clinical trials incorporating epitranscriptomic biomarkers for patient stratification and pharmacodynamic monitoring.

Despite their potential, single-cell epitranscriptomic approaches face technical limitations, including low RNA input requirements, amplification biases, and computational complexity. Continued methodological refinement, protocol standardization, and development of accessible bioinformatic pipelines are required to fully realize the translational potential of these technologies. Systematically bridging the gap between basic science discovery and clinical application will enable the therapeutic exploitation of epitranscriptomic modulation in wound care.

## Conclusions

Studies of RNA modifications in wound healing expand conventional molecular frameworks, establishing epitranscriptomics as a critical yet underexplored regulator of tissue regeneration. Several key concepts emerge. First, RNA modifications function as dynamic molecular switches, enabling rapid post-transcriptional reprogramming during repair and challenging purely genetic models of regulation. Second, epitranscriptomic marks—particularly m^6^A—serve as an interface between metabolic dysfunction and impaired regeneration, as illustrated by diabetic wounds, where the m^6^A landscape acts as a molecular sensor of metabolic stress. Third, selective targeting of RNA modification pathways offers promise for precision therapies in refractory wounds, contingent upon careful, phased clinical evaluation. Advancing the field requires deciphering the epitranscriptomic code using advanced methodologies, including single-cell RNA modification sequencing and CRISPR-based editing, to develop novel therapeutic strategies. Significant translational challenges remain, encompassing the development of targeted delivery systems, elucidation of cell-type-specific modification dynamics, and rigorous clinical validation of epitranscriptomic interventions. Interpretation must account for spatiotemporal heterogeneity and employ site-resolved mapping, orthogonal validation, and phase-matched perturbation approaches to distinguish causation from correlation, as neglecting these considerations may result in overestimation of therapeutic efficacy or compromised translational applicability to human wounds. Ultimately, robust validation across models—from two-dimensional cultures and three-dimensional organoids to rodent, large-animal, and human *ex vivo* systems—is essential, with careful attention to species differences, technical limitations, and causal specificity. Only by meeting these criteria can epitranscriptomic therapies achieve meaningful clinical impact. In summary, RNA modifications extend beyond their biochemical functions to redefine the understanding of cellular plasticity and tissue regeneration. This review decodes the intricate molecular language of RNA modifications, providing a foundation for personalized and precision wound healing strategies capable of improving outcomes across diverse clinical contexts.

## Abbreviations

ac^4^C: *N*^4^-acetylcytidine; AGEs: advanced glycation end products; ALKBH5: AlkB homolog 5; ALP: alkaline phosphatase; CEWH: corneal epithelial wound healing; circRNAs: circular RNAs; DFUs: diabetic foot ulcers; ECM: extracellular matrix; EPCs: endothelial progenitor cells; FTO: fat mass and obesity-associated protein; hBMSCs: human bone marrow mesenchymal stem cells; HCECs: human corneal epithelial cells; HNRNPA2B1: heterogeneous nuclear ribonucleoprotein A2/B1; HSP70: heat shock protein 70; IGF2BPs: insulin-like growth factor 2 mRNA-binding proteins; iNOS: inducible nitric oxide synthase; lncRNAs: long non-coding RNAs; LNPs: lipid nanoparticles; m^1^A: *N*^1^-methyladenosine; m^5^C: 5-methylcytosine; m^6^A: *N*^6^-methyladenosine; m^7^G: *N*^7^-methylguanosine; METTL1: methyltransferase-like 1; METTL14: methyltransferase-like protein 14; METTL3: methyltransferase-like protein 3; miRNAs: micro RNAs; mRNA: messenger RNA; MSCs: mesenchymal stem cells; NAT10: N-acetyltransferase-like protein 10; ncRNAs: non-coding RNAs; RBM15: RNA-binding motif protein 15; ROS: reactive oxygen species; rRNAs: ribosomal RNAs; SAM: S-adenosylmethionine; snRNAs: small nuclear RNAs; snoRNAs: small nucleolar RNAs; TFPI-2: tissue factor pathway inhibitor-2; tRNAs: transfer RNAs; UVB: Ultraviolet B; WHO: World Health Organization; WTAP: Wilms’ tumor 1 associated protein; YBX1: Y-box binding protein 1; ZC3H13: zinc finger CCCH domain-containing protein 13.
